# Dose-dependent improvement of cardiac function in a swine model of acute myocardial infarction after intracoronary administration of allogeneic heart-derived cells

**DOI:** 10.1186/s13287-019-1237-6

**Published:** 2019-05-31

**Authors:** Veronica Crisostomo, Claudia Baez, José Luis Abad, Belén Sanchez, Virginia Alvarez, Rosalba Rosado, Guadalupe Gómez-Mauricio, Olivier Gheysens, Virginia Blanco-Blazquez, Rebeca Blazquez, José Luis Torán, Javier G. Casado, Susana Aguilar, Stefan Janssens, Francisco M. Sánchez-Margallo, Luis Rodriguez-Borlado, Antonio Bernad, Itziar Palacios

**Affiliations:** 1 Fundación Centro de Cirugía de Mínima Invasión Jesús Usón, Carretera N-521, km 41, 10071 Cáceres, Spain; 2 CIBERCV, Instituto de Salud Carlos III. C/Monforte de Lemos 3-5, Pabellón 11. Planta 0, 28029 Madrid, Spain; 3 Coretherapix S.L.U./Tigenix Group C/Marconi 1, 28076, Tres Cantos, Madrid Spain; 4 Department of Cardiovascular Medicine, UZ Leuven Campus Gasthuisberg, Herestraat 49, B-3000 Leuven, Belgium; 5 Department of Immunology and Oncology, Spanish National Center for Biotechnology (CNB-CSIC), C/Darwin, 3 (Campus UAM Cantoblanco), 28049 Madrid, Spain

**Keywords:** Cardiac progenitor/stem cells, CPC, Acute myocardial infarction, Swine model, Allogeneic, Intracoronary administration

## Abstract

**Background:**

Allogeneic cardiac-derived progenitor cells (CPC) without immunosuppression could provide an effective ancillary therapy to improve cardiac function in reperfused myocardial infarction. We set out to perform a comprehensive preclinical feasibility and safety evaluation of porcine CPC (pCPC) in the infarcted porcine model, analyzing biodistribution and mid-term efficacy, as well as safety in healthy non-infarcted swine.

**Methods:**

The expression profile of several pCPC isolates was compared with humans using both FACS and RT-qPCR. ELISA was used to compare the functional secretome. One week after infarction, female swine received an intracoronary (IC) infusion of vehicle (CON), 25 × 10^6^ pCPC (25 M), or 50 × 10^6^ pCPC (50 M). Animals were followed up for 10 weeks using serial cardiac magnetic resonance imaging to assess functional and structural remodeling (left ventricular ejection fraction (LVEF), systolic and diastolic volumes, and myocardial salvage index). Statistical comparisons were performed using Kruskal-Wallis and Mann-Whitney *U* tests. Biodistribution analysis of ^18^F-FDG-labeled pCPC was also performed 4 h after infarction in a different subset of animals.

**Results:**

Phenotypic and functional characterization of pCPC revealed a gene expression profile comparable to their human counterparts as well as preliminary functional equivalence. Left ventricular functional and structural remodeling showed significantly increased LVEF 10 weeks after IC administration of 50 M pCPC, associated to the recovery of left ventricular volumes that returned to pre-infarction values (LVEF at 10 weeks was 42.1 ± 10.0% in CON, 46.5 ± 7.4% in 25 M, and 50.2 ± 4.9% in 50 M, *p* < 0.05). Infarct remodeling was also improved following pCPC infusion with a significantly higher myocardial salvage index in both treated groups (0.35 ± 0.20 in CON; 0.61 ± 0.20, *p* = 0.04, in 25 M; and 0.63 ± 0.17, *p* = 0.01, in 50 M). Biodistribution studies demonstrated cardiac tropism 4 h after IC administration, with substantial myocardial retention of pCPC-associated tracer activity (18% of labeled cells in the heart), and no obstruction of coronary flow, indicating their suitability as a cell therapy product.

**Conclusions:**

IC administration of allogeneic pCPC at 1 week after acute myocardial infarction is feasible, safe, and associated with marked structural and functional benefit. The robust cardiac tropism of pCPC and the paracrine effects on left ventricle post-infarction remodeling established the preclinical bases for the CAREMI clinical trial (NCT02439398).

**Electronic supplementary material:**

The online version of this article (10.1186/s13287-019-1237-6) contains supplementary material, which is available to authorized users.

## Background

Heart-derived stem/progenitor cells (CSC/CPC) have been reported to improve functional recovery after myocardial infarction in large animal preclinical studies. The two main heart-derived cell populations have been previously studied: c-kit+ CPC [1–3] and cardiosphere-derived cells (CDC) [4–6]. These studies suggest that heart-derived cells could have a potential therapeutic capacity to reduce the burden of heart disease, still the number one cause of death worldwide [7]. Several groups have reported encouraging results in the first clinical trials conducted with autologous CSC/CPC in both ischemic and non-ischemic cardiomyopathy [8, 9].

The beneficial effects of CSC/CPC were initially attributed to their potential to engraft and differentiate towards different cell types. There is nonetheless insufficient data of in vivo transdifferentiation of transplanted CSC/CPC into relevant numbers of functional reparative cells in injured tissues [10, 11]. There is a growing body of evidence supporting that tissue repair is predominantly mediated by paracrine factors or extracellular particles secreted by CSC/CPC. This complex combination of secreted factors promotes survival of myocardial cells at risk and stimulates neovascularization, resulting in durable benefits despite the short survival of transplanted cells [11–14].

The use of an allogeneic therapy could expand CSC/CPC indications to acute myocardial infarction (AMI). Allogeneic cardiac-derived cell products offer a readily available, off-the-shelf alternative that can therefore be administered early after the ischemic event, opening the door to therapy in the acute stage. This possibility has been tested in a variety of preclinical studies and indications [1, 6, 15] that recapitulate the pioneering experience with mesenchymal stem cells [16]. Allogeneic approaches obviate the need for endomyocardial biopsies, allow better quality control of cell production (from donor’s screening to pre-freezing and post-thawing sterility and viability), and minimize the potential inter-individual variability and expansion failures of autologous cell cultures. The positive impact on production costs may also facilitate future clinical introduction of this therapy into a clinical routine [17]. Moreover, autologous stem cells harvested from cardiac patients may have compromised regenerative capacity, because they have been subjected to the same risk factors associated with ischemic heart disease. To elucidate the importance of these concerns, a recent meta-analysis has compared autologous and allogeneic cell therapy in heart disease, finding a similar effectivity from both cell types, which leads the authors to recommend an allogeneic focus for future trials based on the logistical advantages of these cells [17, 18].

Human c-kit+ CPC (hereafter referred to as CPC) have been previously characterized as a mesenchymal stem cell (MSC)-like population [19] with a significant Treg-mediated immunomodulatory capacity when introduced in an inflammatory environment, such as the one encountered immediately after AMI [19, 20]. In addition, the retention of allogeneic CPC is enhanced through interaction with NK cells [21]. Together, these two features represent an additional potential advantage of allogeneic over autologous CPC therapy.

Preliminary clinical trials [22, 23] conducted with allogeneic cardiac-derived cell products for AMI have reported robust safety and feasibility of this therapy, the primary endpoint in a small STEMI trial [24], but have failed to show effectivity.

Finally, while extensive dose-response studies have been performed using MSC in clinical trials [25, 26], quantitative data with heart-derived cell products are scarce and limited to preclinical trials [15, 27, 28]. Rodent studies identified a therapeutic range with a flat dose-response, with low doses being ineffective and high doses proving harmful [27]. Large animal studies report that intracoronary CDC optimal dose lies within the 7.5 to 10 million cells range [28]. Our prior results administering 25 × 10^6^ cells via the infarct-related artery in absence of toxicity [1] support the preclinical evaluation of a higher dose performed in the present work.

In terms of cell dosage, 25 × 10^6^ and 50 × 10^6^ pCPC were selected considering cell sizes and taking as a reference previous works with CDC. CDCs are reported to be ∼ 20 μm in diameter [5, 28], and the maximum dose of CDC that can be safely administered in acutely infarcted swine is 12.5 × 10^6^ [28]. However, pCPC used in this study are between 13 and 14 μm in diameter [1]; so, we hypothesized that a greater amount of cells could be administered safely. Moreover, any signal of danger during injection would be detected in the present work.

We therefore used a large animal model to investigate homing of radiolabeled allogeneic porcine cardiac cell (pCPC) populations in the ischemic myocardium and to explore dose-dependent effects on structural and functional infarct remodeling using serial, comprehensive MRI imaging. These pCPC have been obtained and expanded following equivalent protocols as their human counterparts (CPC) [20, 21]. Our results indicate that pCPC administration via the infarct-related coronary artery, at a previously defined optimal time window [1], is safe and associated with beneficial dose-dependent functional and structural improvement in the infarcted porcine heart.

## Methods

### Isolation and culture of pCPC

The isolation and culture of swine CPC (pCPC) were performed as previously reported [1]. pCPC were expanded over three passages (Additional file 1) and then cryopreserved in a medium with 5% of dimethyl sulfoxide (DMSO). A second expansion was performed to obtain the final product, used for in vivo administration.

### Characterization of pCPC

pCPC were characterized by flow cytometry, real-time quantitative PCR (RT-qPCR), and enzyme-linked immunosorbent assay (ELISA). The migration-promoting capacity of conditioned medium (CM) was also evaluated. These procedures are detailed in Additional file 1.

#### Flow cytometry

The expression of pCPC surface markers was analyzed by flow cytometry, using the antibodies indicated in Additional file 1.

#### RT-qPCR and genomic PCR

Total RNA was isolated from two different batches of pCPC (pCPC01 and 03). Reverse transcription quantitative PCR (qPCR) was carried out using TaqMan probes (Invitrogen) in triplicate for each sample and each gene. For the detection of male swine genomic sequences (Y chromosome), genomic DNA PCR was carried out using previously described primers and indicated conditions.

#### ELISA

Using pCPC- and CPC-conditioned medium prepared in parallel with the concentrations of monocyte chemoattractant protein 1 (MCP-1 or CCL2), insulin-like growth factor 1 (IGF-1), transforming growth factor β1 (TGF-β1), stromal-derived factor 1α (SDF-1α or CXCL12), and hepatocyte growth factor (HGF) were measured with ELISA kits (R&D Systems Inc., Minneapolis, MN), according to the manufacturer’s instructions.

#### Transwell migration assay

pCPC- and CPC-conditioned media were evaluated, following standard methods, for their capacity to induce migration of MonoMac-1 cell line (DSMZ) in Boyden chambers with 5 μm pores. The migration index was calculated as the ratio between the number of migrated cells in response to different stimuli (mean of the duplicates minus background) and the cells migrated in the absence of a stimulus (background).

#### ^18^F-FDG labeling of pCPC

^18^F-FDG uptake was optimized for labeling 2% of the total cell dose to be administered. Aliquots of 0.5 × 10^6^ cells were labeled using a dosage of 100 μCi of ^18^F-FDG by incubation 60 min at 37 °C in glucose-free Dulbecco modified Eagle medium (DMEM) supplemented with 5% human serum albumin. Cells were then washed twice with phosphate-buffered saline (PBS) and resuspended in warm DMEM for implantation. Supernatant and pellet (cells) radioactivity were measured in a dose calibrator. A trypan blue viability test was performed to calculate cell viability before and after radiolabeling.

### Large animal model experimentation

**Fig. 1:**
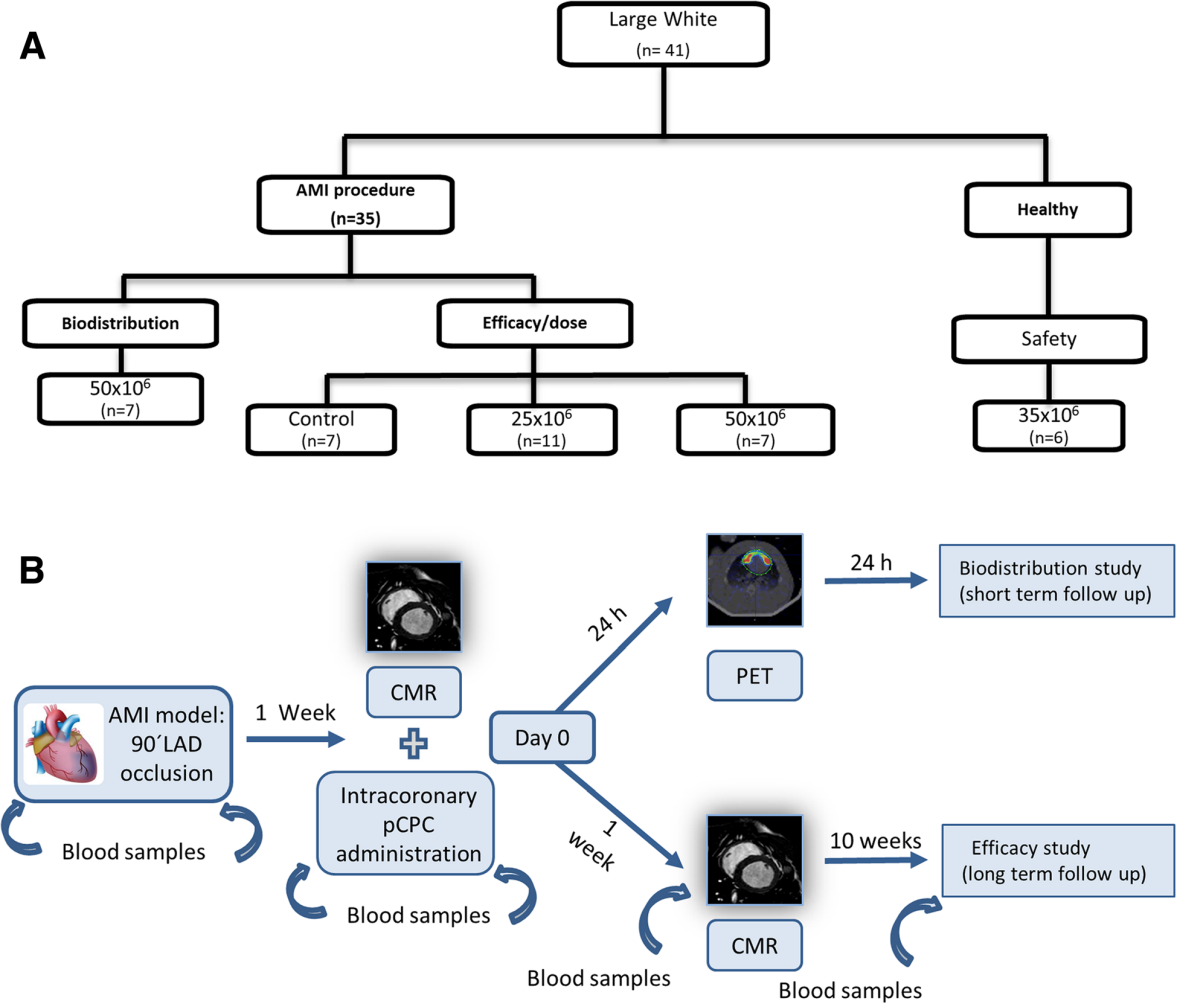
Large animal studies. Experimental workflow. **a** Flow chart illustrating the study design in large white swine. **b** AMI induction, treatment and sacrifice timetable, AMI indicates acute myocardial infarction. pCPC, cardiac stem/progenitor cells isolated from large white swine. LAD, left anterior descending coronary artery; CMR, cardiac magnetic resonance; PET, positron emission tomography

A total of 28 infarcted female large white swine were used for the dose-response study, 6 healthy swine for the acute safety, and 7 additional infarcted swine for biodistribution analysis. The studies performed are summarized in Fig. 1 and detailed in Additional file 1 (Fig. 1a, b).

### Statistical analysis

Data are presented as means ± standard deviations. Differences between the groups were identified and compared using the Kruskal-Wallis and Mann-Whitney *U* tests, and intragroup comparisons were performed with the Wilcoxon paired samples test. Values of *p* < 0.05 were considered significant. All *p* values were the results of two-tailed tests. Calculations were performed using the SPSS 18.0 statistical package for Windows (SPSS Inc., Chicago, IL).

## Results

### Comparative characterization of swine cardiac progenitor cells

pCPC were isolated and expanded similarly to their human counterparts tested in the CAREMI clinical trial [1, 23]. To ensure genetic stability in pCPC, karyotype analyses were performed at different moments of the expansion process. These studies did not reveal any significant alteration in genetic stability (Additional file 1: Figure S1A). The same result was obtained for human CPC by comparative genomic hybridization (CGH), as previously reported [23].

**Fig. 2:**
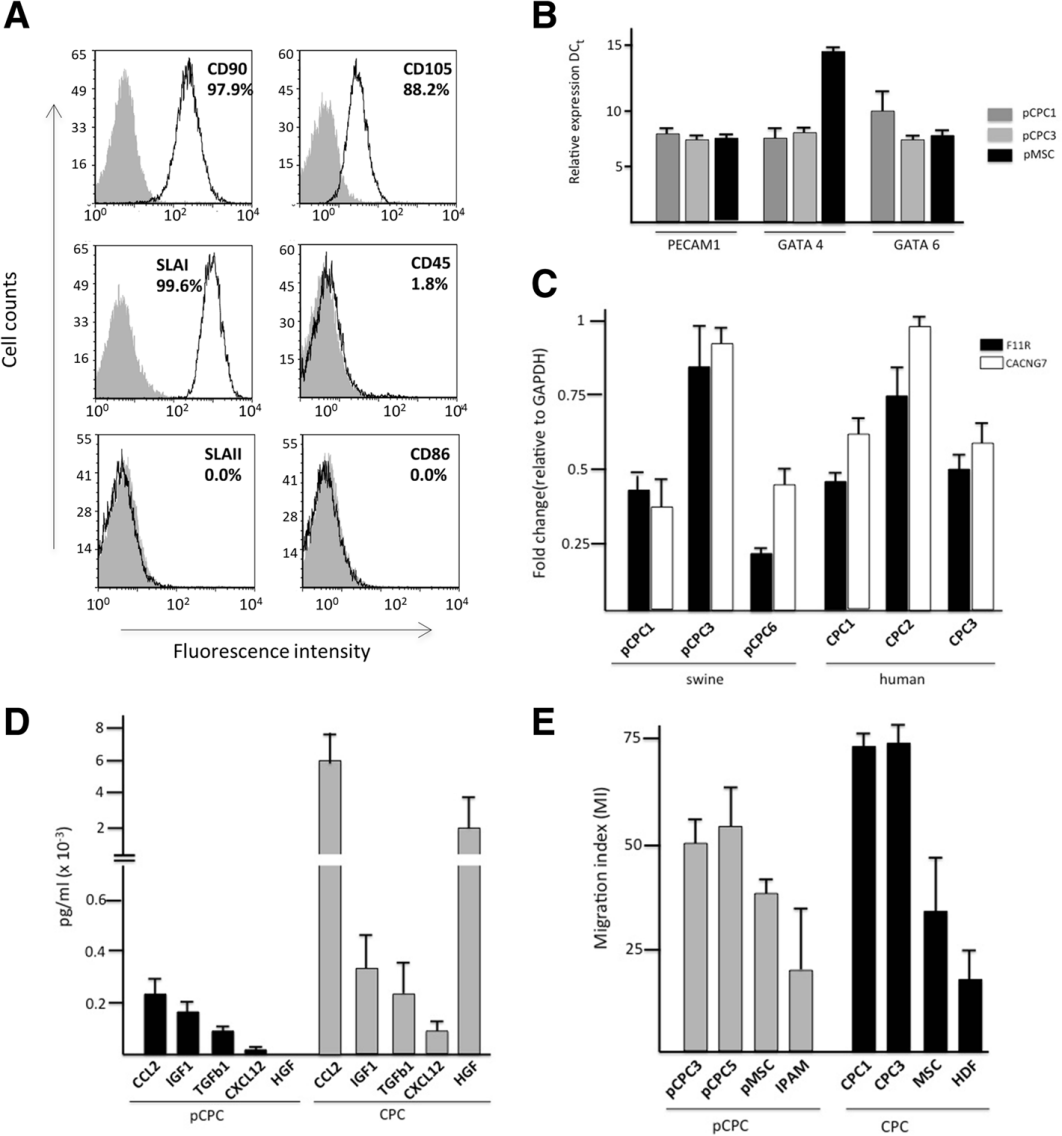
Phenotypic and functional characterization of pCPC. Comparison with hCPC. **a** Swine CPC characterization by flow cytometry. Expression of CD90, CD105, CD45, SLAI, SLAII, and CD86 is shown (empty histogram) and the number of positive cells is indicated (%). Gray-filled area represents isotype control. **b** RT-qPCR analysis of PECAM1 (CD31), GATA4, and GATA6 expression in the pCPC batches. Ct value for each sample/gene analyzed. There are no significant differences between the batches used. The average expression normalized to beta-2-microglobulin (β2M) is shown. Error bars represent SD (*n* = 3). **c** Comparative expression analysis of F11R and CACNG7 membrane makers, in both swine and human isolates; three independent isolates were compared for each cell type. The assay was performed three times, and data are expressed as mean ± SD; black lines indicate the *p* value summary (***< 0.002, **< 0.02, *< 0.05) (one-way analysis of variance followed by the Bonferroni multiple comparison test). **d** Porcine CPC (*n* = 4) secretome characterization by ELISA compared to human CPC (*n* = 3) secretome. The results are expressed as mean ± SD in pg/mL. **e** Migration assay. Conditioned medium (CM) of human cells (CPC1, CPC3, MSC, and HDF), were compared with CM obtained from swine samples (pCPC3 and pCPC5, pMSC and IPAM (pig alveolar macrophages) in their capacity to trigger the migration of MonMac-1 cells

Cytometric analysis of several pCPC isolates (Fig. 2a) showed positive expression of CD90 (Thy1), CD105 (endoglin), low expression for swine leukocyte antigen class I (SLA-I), and negative for CD45, CD86, and swine leukocyte antigen class II SLA-II. Moreover, pCPC do not express CD34 nor CD31 (PECAM), recapitulating the profile described for human CPC [1, 14, 19, 20, 24]. An extended characterization is shown in Additional file 1 for four batches of pCPC and mesenchymal stem cells from bone marrow (BM-MSC) or adipose tissue (ADSC) (Additional file 1: Figure S1B). Several markers are common and highly expressed in all cell types: CD29, CD44, CD90, and CD105 whereas others including CD40, SLA-II, and CD86 are barely expressed. CD31, GATA 4, and GATA 6 genes were confirmed by RT-qPCR showing similar results to those obtained with the CPC from human origin (Fig. 2b).

Based on complementary previous analyses, both with CPC [29] and pCPC (Prat et al. 2019, in preparation), an important global similarity between porcine-derived CPC and human-derived CPC was established. We then validated some of the array-based findings analyzing by RT-qPCR expression of F11R (F11 receptor) and CACNG7 (calcium channel, voltage-dependent, gamma subunit 7), both proposed as CPC markers [29]. Both genes demonstrated a similar expression profile, by RT-qPCR, in pCPC and CPC (Fig. 2c), although with more variable expression levels between pCPC isolates.

Increasing evidence supports that current cell therapy approaches improve cardiac function mainly via paracrine mechanisms, with extracellular particles playing an important role [30, 31]. In order to provide evidences of a substantial functional analogy of pCPC with hCPC, we first evaluated their secretome, preparing a selection of cytokines that have been demonstrated to be present in hCPC secretome at higher levels than in MSC- and HDF-conditioned medium [14]. We thus compared by ELISA the production of five proteins by both hCPC and pCPC, specifically two chemokines (CCL2 and CXCL2), two growth factors (IGF-1 and HGF), and one cytokine (TGFb1).

Human CPC secreted significantly higher amounts of CCL2, IGF-1, TGFβ1, CXCL12, and HGF than pCPC (Fig. 2d); however, the absolute differences may be accounted for by the use of human-specific ELISA kits and suboptimal cross-reactivity in pigs.

Finally, we evaluated the capacity of pCPC to stimulate migration of MonMac-1 cells using conditioned medium (pCPC-CM) of two different cell lines (pCPC3 and pCPC5). This activity is related to the capacity of pCPC to produce and secrete the aforementioned chemokines. pCPC-CM was compared to CM obtained from human CPC (CPC1, CPC3), MSC, human dermal fibroblast (HDF), pMSC from adipose tissue, and immortalized pig alveolar macrophages (IPAM) (Fig. 2e). pCPC-CM promoted greater migration than pMSC-CM or IPAM. However, the activity was lower than that observed with human CPC-CM.

In summary, these results show that pCPC appear to have a weak immunogenic profile with negative expression of SLA-II and low expression of SLA-I (equivalent to HLA expression in human CPC), which may support their in vivo application in allogeneic setting. Additionally, phenotypic analysis of surface membrane markers, genes expression, and migration assays in pCPC show strong similarities to their human counterparts.

### Safety and biodistribution analyses after intracoronary pCPC administration in infarcted swine

**Fig. 3:**
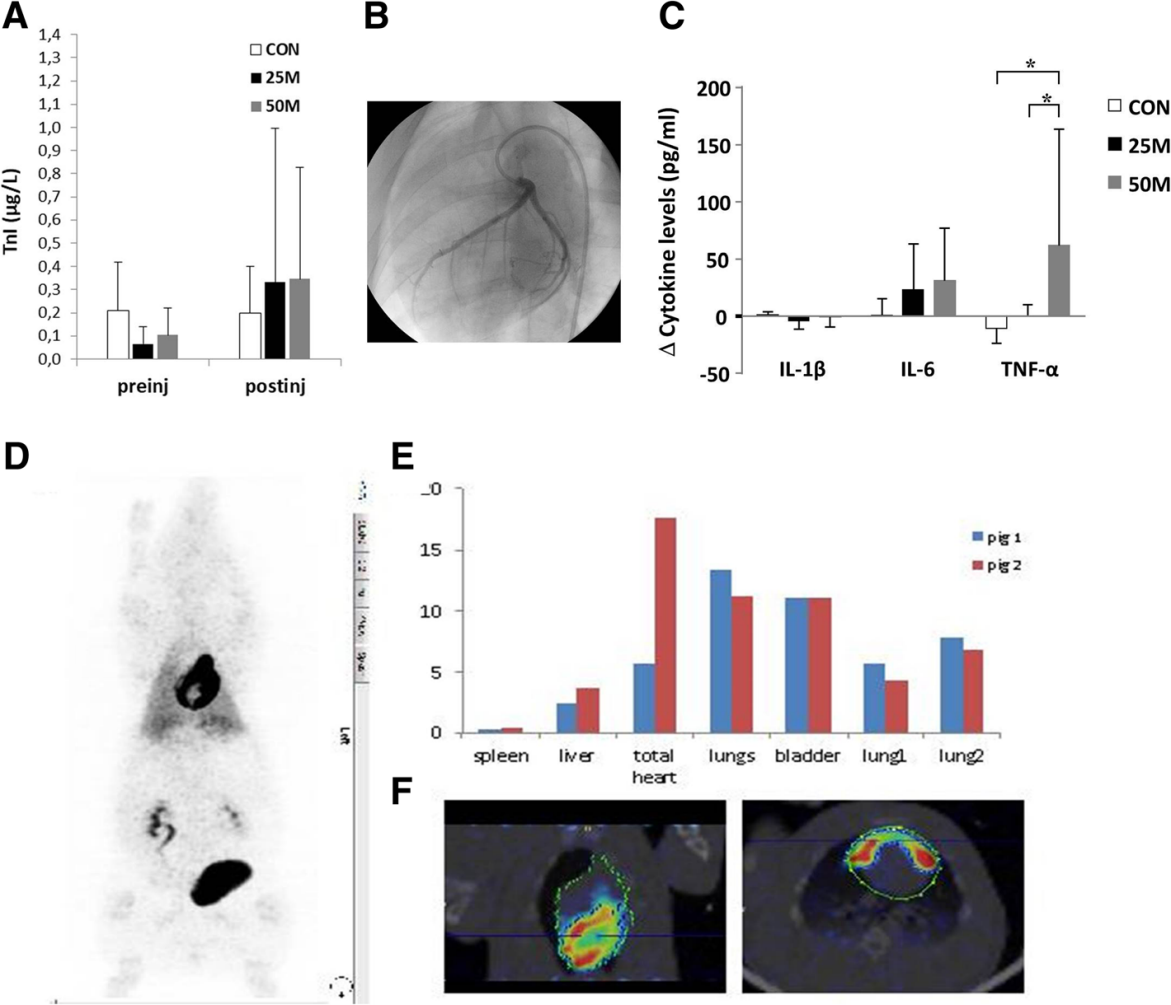
Acute toxicity and biodistribution of pCPC in infarcted swine. **a** cTnI values measured over the course of the study. The slight elevation seen 24 h after vehicle/cell administration was not significantly different between groups. **b** Coronariogram obtained immediately after pCPC administration in a 50-M animal depicting complete opacification of the artery (TIMI 3). **c** Cytokine levels measured in plasma samples. Bars show the differences at 24 h after vehicle/cell administration referred to pre-administration values. **d** PET/CT images after ^18^F-FDG-labeled CPC administration. pCPC labeled with ^18^F-FDG were intracoronary administered in pigs 1 week after infarction. Cell distribution was analyzed by PET 4 h after cell infusion. **d** PET maximal intensity projection (MIP) images, showing the distribution of ^18^F-FDG activity over the entire body of the animal. **e** ^18^F-FDG activity could also be clearly detected in the bladder (b), kidneys (k), and lungs (l). **f** Sagittal sections of PET/CT images only in the heart area; a diffuse uptake is shown

The intracoronary (IC) administration of different doses of allogeneic pCPC (25 × 10^6^cells, *n* = 11 [25 M group]; 50 × 10^6^ cells, *n* = 7 [50 M group]) or vehicle (*n* = 7; CON) was analyzed. Three animals died during infarct induction due to refractory arrhythmias. Infarction was successfully induced in 25 surviving animals, as demonstrated by increased cTnI values 24 h after balloon inflation. No differences were seen between the groups in any MR-derived parameters on day 0 (pre-injection, Table 1), thus confirming that both AAR and infarct sizes (which ranged from 23.4 to 27.3% and from 13.4 to 16.7%, respectively), and their effects on functional parameters (LVEF, EDVi, and ESVi) were comparable in all groups prior to pCPC or vehicle injection. Administration of pCPC or vehicle was performed 7 days after infarction, without major adverse cardiac events during or after injection in any group (Fig. 3a, b). Moreover, slight increases in cTnI were observed in the treated groups after intervention (Fig. 3a) but remained within a clinically acceptable range. One animal belonging to group 50 M showed TIMI2 coronary flow after injection but recovered after nitroglycerine administration (400 μg). One animal belonging to the 25 M group died during follow-up, 4 weeks after pCPC injection. Necropsy in this animal did not show significant lesions in any organ; no cause of death could be identified.

**Table 1:** Main cardiac parameters calculated from magnetic resonance exams performed throughout the study

Groups	CON (vehicle)				25 M (25 × 10^6^ pCPC)				50 M (50 × 10^6^ pCPC)			
	Day 7 (healthy)	Day 0 (preinjection)	1 week	10 weeks	Day 7 (healthy)	Day 0 (preinjection)	1 week	10 weeks	Day 7 (healthy)	Day 0 (preinjection)	1 week	10 weeks
LVEF (%)	54.9 ± 11.2	38.0 ± 9.6	40.4 ± 5.4	42.1 ± 10.0	52.5 ± 6.4	41.7 ± 8.1	42.8 ± 7.6	46.5 ± 7.4	52.0 ± 6.2	40.1 ± 6.5	43.7 ± 6.5	50.2 ± 4.9*
EDVi (mL/m^2^)	80.0 ± 18.4	105.0 ± 10.6	111.7 ± 11.2	119.0 ± 24.8	80.1 ± 11.7	97.2 ± 9.7	108.6 ± 15.3	104.3 ± 14.2	84.1 ± 10.1	94.8 ± 14.7	97.5 ± 17.6	94.0 ± 11.4**
ESVi (mL/m^2^)	36.5 ± 12.7	65.4 ± 14.5	66.7 ± 10.5	70.5 ± 25.5	38.3 ± 8.5	56.7 ± 9.2	62.8 ± 15.3	56.3 ± 14.0	40.4 ± 7.3	56.7 ± 11.9	56.2 ± 17.8	47.0 ± 8.2**
Infarct (%)	n/a	16.7 ± 5.1	11.3 ± 2.7	8.3 ± 2.8*	n/a	14.6 ± 6.1	11.0 ± 6.7	8.7 ± 5.2	n/a	13.4 ± 5.3	11.0 ± 5.4	5.9 ± 4.1*
edema (%)	n/a	23.4 ± 5.9	18.7 ± 4.4	6.7 ± 1.1	n/a	25.2 ± 6.6	14.2 ± 5.4	6.7 ± 3.9	n/a	27.3 ± 8.4	15.0 ± 4.2	5.3 ± 3.6

Data presented as mean ± standard deviation. Infarct area is expressed as % of the left ventricle. Edema was calculated at a mid-heart slice and expressed as % of this slice

*LVEF* left ventricular ejection fraction, *EDVi* end-diastolic volume indexed to body surface area, *ESVi* end-systolic volume indexed to body surface area, *n/a* not applicable

*p < 0.05 compared to preinjection (day 0) values within groups

**p < 0.05 compared to CON at the same time point

Measurements of plasma cytokines revealed that six out of nine cytokines (i.e., IFNγ, IL-4, IL-8, IL-10, and IL-12p40) were undetectable, which could be related to the detection limit of commercially available swine immune reagents. In contrast, IL-1β, IL-6, and TNF-α levels were detected. No significant difference was found for IL-1β in any of the groups. However, the pro-inflammatory cytokines IL-6 and TNF-α were significantly increased 24 h after infusion of 50 × 10^6^ allogeneic pCPC. In contrast, intracoronary administration of vehicle (CON group) and 25 × 10^6^ pCPC did not cause any significant changes in these pro-inflammatory cytokines over time (Fig. 3c and Additional file 1: Table S1).

To study cell homing and biodistribution in the injured myocardium, a separate group of infarcted animals (*n* = 7) received an IC infusion of ^18^F-FDG-labeled pCPC (50 × 10^6^cells/animal) and biodistribution analysis was performed using PET-CT at 4 h after administration (Fig. 3d–f). PET results indicated that a substantial fraction (18%) of the transferred ^18^F-FDG-labeled pCPC was detected in the heart, followed by uptake in the bladder (11%), lungs (4–6%), and liver (4%) and low level in the spleen (< 1%). These data show cardiac tropism, coronary clearance, and substantial myocardial tissue retention of pCPC, indicating their safety and suitability for coronary injection.

### Dose-response after IC pCPC administration in infarcted swine

**Fig. 4:**
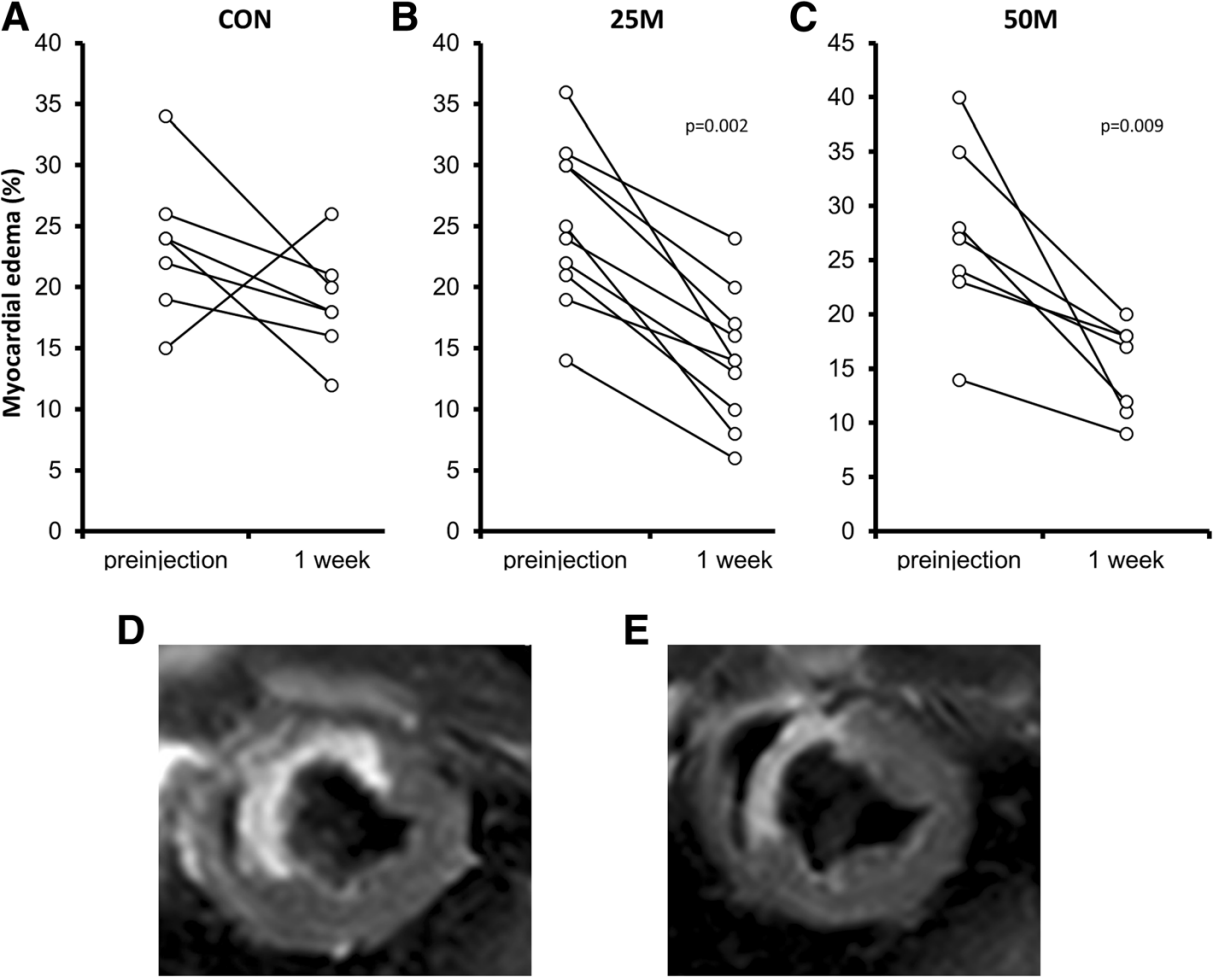
Evaluation of early edema 1 week after the treatment. Mean edema percentage before and 1 week after pCPC administration in animals receiving (**a**) vehicle (CON), (**b**) 25 × 10^6^ pCPC (25 M), or (**c**) 50 × 10^6^ pCPC (50 M). **d** Short-axis images of a mid-ventricular slice acquired pre-injection and **f** 1 week post-injection in a representative animal belonging to the 50 M group. *p* values obtained using non-parametric tests (Mann-Whitney *U* test)

Cardiac function parameters derived from CMR studies are presented in Table 1. Interestingly, early after transplantation, there was a significant decrease in edema in both treatment groups (Fig. 4) 1 week after pCPC injection (the percentage of edema at a mid-ventricular slice decreased from 27.3 ± 8.4% to 15.0 ± 4.2%, *p* = 0.009, in 50 M animals; from 25.2 ± 6.6% to 14.2 ± 5.4%, *p* = 0.002, in 25 M animals). In contrast, in CON animals, this change was not significant (edema decreased from a pre-injection value of 23.4 ± 5.9% to 18.7 ± 4.4%, *p* = 0.128).

**Fig. 5:**
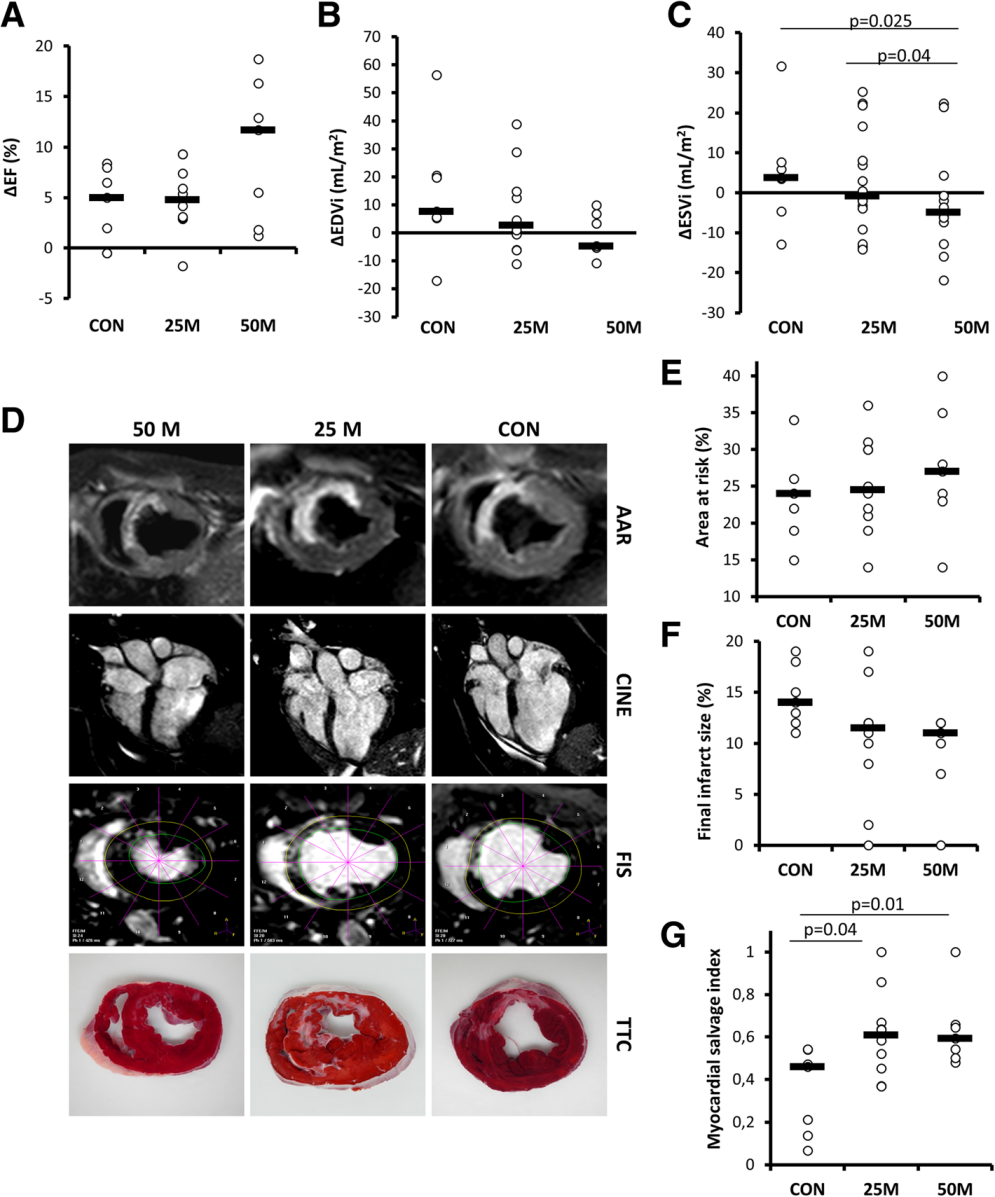
Evolution of myocardial damage parameters after pCPC administration in infarcted swine. **a**–**c** Changes over time in cardiac function parameters as measured with cardiac magnetic resonance (CMR) for the three experimental groups (CON, vehicle; 25 M, receiving 25 × 10^6^ pCPC; 50 M, receiving 50 × 10^6^ pCPC). Treatment effects (defined as the difference between pre-injection and 10-week values). **a** Changes in left ventricular ejection fraction (LVEF). **b** End-diastolic volume indexed to body surface area (EDVi). **c** End-systolic volume indexed to body surface area (ESVi). **d** Representative CMR and TTC-stained slices from the three studied groups. **e** Myocardial edema/area at risk (AAR) was calculated as a percentage of the left ventricle in a mid-heart slice using T2-weighted imaging. **f** Final infarct size (FIS) in an equivalent slice. **g** Myocardial salvage index (MSI) was then computed as AAR at mid-heart slice minus FIS in an equivalent slice divided by AAR (MSI = (AAR-FIS)/AAR). *p* values obtained using non-parametric tests (Kruskal-Wallis and Mann-Whitney *U* tests)

The analysis of the evolution of the studied cardiac function parameters (Table 1) indicated significant decreases in LVEF from baseline to day 0 (7 days after infarction and just before IC pCPC or vehicle injection), followed by a progressive recovery from this time point to 10 weeks, that proved significant only in the 50 M group (preinjection 40.5 ± 6.54%, 10 weeks 50.2 ± 4.9%, *p* = 0.021). Similarly, ventricular volumes increased significantly from baseline to 1 week after myocardial infarction in all groups, followed by a trend towards recovery in both cell treatment groups, so that in the 50 M group, EDVi and ESVi at 10 weeks were not significantly different (*p* = 0.128) from pre-infarction values, while dilatation further increased in CON animals. Treatment effects (defined as the difference between pre-injection and 10 weeks values) are shown in Fig. 5a–c. The change in LVEF was much greater in 50 M animals compared to 25 M and CON (ΔLVEF was 9.7 ± 6.9% in 50 M, 4.9 ± 3.4% in 25 M, and 4.1 ± 3.8% in CON, as shown in Fig. 5a). The changes in LVEF in the 50 M group were accounted by a trend towards less dilatation (Fig. 5b) but most predominantly by a significant (*p* = 0.025) decrease in ESVi with volumes approaching pre-infarction ranges (Fig. 5c). Interestingly, the magnitude of ESVi changes over this period showed improvements in both treated groups, reflecting also a slight reduction in the 25 M group.

Myocardial salvage index (MSI, Fig. 5g), obtained from area-at-risk measurements on T2W images (Fig. 5e) and final infarct size determinations on corresponding mid-ventricular slices (Fig. 5f), was 0.35 ± 0.20 in CON, significantly lower than in treatment groups (MSI = 0.61 ± 0.20, *p* = 0.04, in 25 M; MSI = 0.63 ± 0.17, *p* = 0.01, in 50 M), suggesting a cardioprotective effect of the administered cells (Fig. 5g). This functional improvement, however, was not accompanied by statistically smaller scar sizes (Table 1) when the three groups were compared, despite a clear trend towards smaller infarct sizes in the 50 M group at 10 weeks (5.9% ± 4.1% versus 8.7% ± 5.2% in the 25 M group and 8.3% ± 2.8% in CON). Within groups, however, infarct size decreases were significant in both CON and 50 M groups (*p* < 0.05).

### Assessment of scarred and viable myocardium

After euthanasia, engraftment of transplanted male pCPC cells was inferred from the analysis of Y chromosome sequences (from the administered cells) in female recipient swine. No male sequences were amplified in any of the samples studied at 10 weeks after pCPC treatment (Additional file 1: Figure S2A), suggesting that most of the beneficial lasting effects must be due to the pCPC-mediated paracrine effects and not to the stable engraftment or any derived progeny [11].

**Fig. 6:**
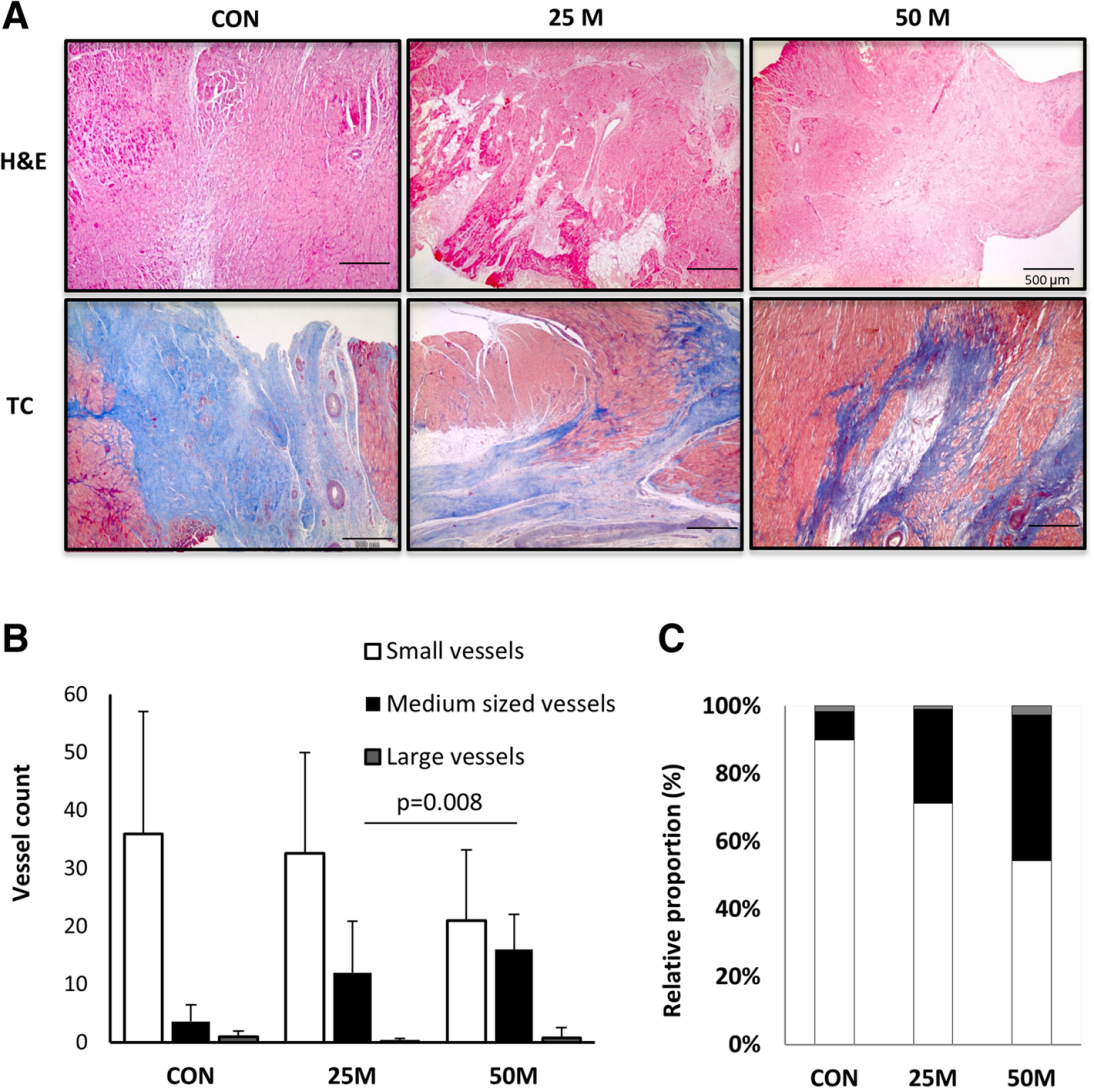
Histopathological studies. **a** Hematoxilin-eosin and Massons trichromic stains show typical histological appearance of the infarcts in control animals with increased collagen, while viable myocardial muscle bundles can be seen in treated animals. The bar represents 500 μm. **b**, **c** Distribution of vessels’ sizes, as determined at the infarct border

Pathological analysis of cardiac samples from all animals found no evidence of teratoma formation in any case (Additional file 1: Figure S2B). Overall, at the time of euthanasia, inflammation was limited, necrosis mild, and calcification mostly absent, except for one CON animal. Interestingly, post-infarction fibrotic scars, which were graded severe in CON [4], were scored as slight [1] or mild [2] in 25 M and 50 M, respectively (*p* = 0.031). In CON swine, this fibrosis constituted a wide, well-formed band, while scar tissue in cell-treated animals was less organized with collagen fibers interspersed with variably sized clusters of viable cardiac myocytes (Fig. 6a).

Finally, morphometric evaluation of angiogenesis between the groups found no differences in the total amount of vessels, but the distribution of vessel sizes was significantly different between the groups (*p* = 0.031, Fig. 6b, c). Post hoc comparisons showed this difference to be related to a greater representation of medium-sized vessels in cell-treated groups, suggesting a more mature angiogenic response compared to CON; the difference was statistically significant (*p* = 0.008) in the 50 M group (Fig. 6b).

### Acute and sub-acute toxicity

**Fig. 7:**
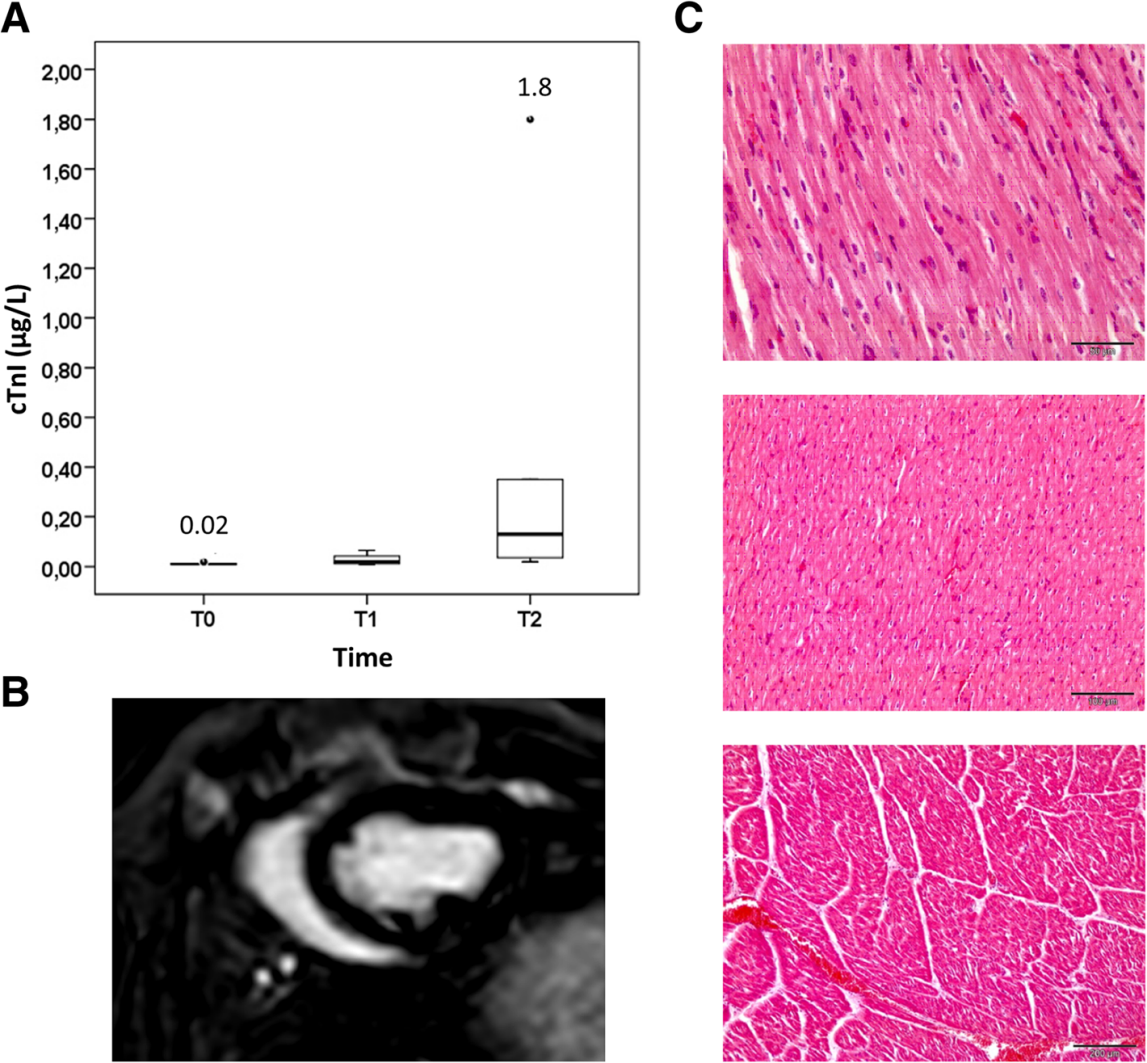
Feasibility and safety study in healthy swine. pCPC (35 × 10^6^) were administered via the LAD in healthy swine (*n* = 7). **a** Changes to cTnI (μg/L) observed at 4 h (T1) and 24 h (T2) after injection. **b** DE-CMR obtained at 24 h and 7 days showed no evidence of infarction. Representative short-axis image obtained 24 h after injection. **c** Hematoxilin-eosin staining of pigs hearts 3 weeks after cell administration. No tissue alterations or inflammatory processes were found in any case

For safety studies, according to the regulatory requirements, healthy non-infarcted female swine received allogeneic pCPC, without any immunosuppressive regimen, in order to assess immediate or short-term adverse effects or toxicity of the administered cells. Animals received 35 × 10^6^ allogeneic pCPC (*n* = 6) via IC infusion, the highest dose of human CPC that was evaluated in the CAREMI clinical trial [23, 24]. Coronary flow was not affected by pCPC infusion (TIMI = 3 in all cases, both before and after cell administration). No ST-segment elevations or cardiac events were observed during injection. cTnI increased slightly after intervention but remained within clinically acceptable values that are attributable to the percutaneous intervention (Fig. 7a). No hyperenhanced areas were identified on follow-up DE-CMR examinations (Fig. 7b). Three weeks after cell administration, histological analysis showed no signs of toxicity (Fig. 7c) or inflammatory process against allogeneic cells, both of which, if present, might suggest a strong immune rejection of infused allogenic cells [4, 32]. These findings in healthy immunocompetent animals further support the safety of IC allogeneic pCPC treatment for acute myocardial infarction.

## Discussion

Myocardial infarction and its associated hemodynamic overload trigger a compensatory left ventricular hypertrophy that eventually evolves into maladaptive remodeling. Although current treatments have dramatically decreased the incidence of AMI-related deaths, paradoxically, this has contributed to an epidemic of chronic ischemic heart disease [7]. Due to the limited success of traditional therapies, research in stem cell-based therapies has intensified as an alternative that could counteract deleterious heart remodeling. Since 2001 [33], numerous pre-clinical and clinical trials have rendered disparate and mostly insufficient results [34]. A report based on individual patient data from several randomized controlled trials concluded no statistically significant benefit, in terms of clinical events or changes in LV function after the IC administration of several modalities of cell therapy [35]. Several factors influencing response must be considered, related not only to the patient’s clinical condition and immunocompatibility profile [19] but also to the manufacturing procedure. For example, the use of low oxygen tensions seems to influence both the yield and the genetic integrity and functionality of expanded cells in different cell lineages [36, 37].

The allogeneic adult heart-derived swine cells used in the present work were isolated, like their human counterparts in the CAREMI clinical trial, by a positive immunomagnetic selection of c-kit. The evolution of c-kit expression was similar to that previously described for human CPC: c-kit was detectable in the first passage in all batches (around 12%), but in later passages (P2-P6), c-kit expression was undetectable. Previously, other authors have reported a similar loss of c-kit expression during cell expansion and culture of cardiac mesenchymal cells [38] and cardiac progenitor cells [39].

Our study was designed to first test bioequivalence of allogeneic pCPC and CPC and second to evaluate safety biodistribution and dose response of pCPC in a randomized preclinical study performed with blinded outcome assessment in a relevant large animal model. First, aiming to limit the deleterious effects of oxidative stress, concomitant to the ex vivo cell expansion [36, 37] (genetic instability, senescence, and reduction of therapeutic properties), pCPC were cultured in forced low O_2_ tension (3%), equivalently to CPC in CAREMI [23, 24]. This protocol renders the required CPC/pCPC numbers with less culture passages (higher duplication rate) and with less signs of aging/senescence [14, 19, 24]. Here, we demonstrate that pCPC show a substantial phenotypic and functional profile similarity to CPC. Therefore, from this perspective, pCPC/CPC are obtained in optimized conditions, probably non-directly comparable with the majority of the other clinical trials.

Furthermore, there is no definitive approach to optimize both survival and biological effect of transplanted cell populations. Preliminary work with pCPC established that the intracoronary injection of allogeneic cardiac progenitor cells (25 × 10^6^) in infarcted pigs is safe, both on the same day or 1 week after experimental infarction, although the biological effects on limitation of left ventricular remodeling were stronger when administration was delayed 1 week after infarct induction [1]. Comparable results have been found using MSC in the rat AMI model [40]. Therefore, we used the same administration scheme for this advanced preclinical study. Interestingly, pCPC administration in infarcted swine induced a clear and significant reduction (45%) of edema in both pCPC-treated groups compared with the CON group, analyzed 1 week after pCPC administration. Myocardial edema has functional relevance because the extent of edema correlates with the transmural extent of infarction, hindering myocyte contractility [41]. Recently, it has been reported, both in humans and swine, that myocardial edema presents a bimodal pattern. The first wave is transient (24 h) and strictly attributable to reperfusion; then, immune infiltration starts and a slower but progressive wave develops peaking 1 week after infarction. It is thought that this second wave parallels the initiation of the healing process [41, 42]. pCPC were administered 1 week after AMI, aiming for a better survival upon transplantation. Based on the recent new view of myocardial edema development and impact, we can conclude that pCPC administration may well interfere with further edema expansion, which could be related to better functional preservation.

The doses of pCPC evaluated in this work (25 M, 50 M) are among the highest reported in cardiac-derived progenitors IC administration in swine, which range from 5 × 10^5^ to 12.5 × 10^6^ [2, 4], although higher amounts have been injected transendocardially (up to 150 × 10^6^ CDC) [15]. It has been always assumed that retention of transplanted cells is more efficient using intramyocardial administration, since the coronary circulation cannot efficiently wash out the cells. Some reports, however, have described an unexpected rapid venous washout, rendering a similar retention by the two methods [43, 44]. Another route that has been proposed to safely administer high cell doses is the intrapericardial administration [45, 46], but few reports have evaluated this approach to date, and mostly in a chronic setting. In this scenario, we opted for IC administration since it does not cause unnecessary heart damage and is widely available in most hospitals. A flat dose-response relationship has been previously described in infarcted rats with IC doses of 0.75–3 × 10^6^ CPC, showing similar functional beneficial effects 35 days after administration [27]. In contrast, we did find a dose-dependent improvement in functional parameters.

Doses greater than 25 × 10^6^ CDC have been reported to be deleterious in prior large animal studies [5], increasing cTnI in a dose-dependent manner. In the present study, we report the beneficial effects of both 25 × 10^6^ and 50 × 10^6^ heart-derived cells. The main difference between that study and ours that could account for the different results lies in the infusion protocol. While Johnston et al. report an optimization of the solution (cells and vehicle) that they are injecting, no mention is made to the infusion protocol. We, on the other hand, use a micro-aggregate filter with a pore size of 40 μ and inject at 2 mL/min during 3 min, followed by a 3-min rest period to allow for cell extravasation. The injection cycle was repeated to the total dose depending on the group. With this infusion protocol, we were able to administer our intended doses without cTnI reaching clinically significant increases, as shown in Fig. 3a (specifically, post-infusion cTnI values were 0.2 ± 0.2 μg/L in CON, 0.3 ± 0.7 μg/L in 25 M, and 0.3 ± 0.5 μg/L in 50 M animals). Moreover, and independently of the dose used, MSI was doubled in both treated groups compared to control animals. MSI has been recently reported to allow for a 46 to 65% decrease in sample size in cardioprotection trials, when compared to infarct size alone [47].

As additional safety criteria, the comparative evaluation of the inflammatory status (24 h before and after pCPC administration) in the three groups (CON, 25 M, 50 M) only showed detectable levels of pro-inflammatory cytokines IL-1β, IL-6, and TNF-α. Of these, only IL-6 and TNF-α were significantly increased after IC administration of 50 × 10^6^ pCPC. This could be considered as a “danger signal” of acute damage, although TIMI flow post-injection seemed to be unaffected by the infusion and plasma cTnI levels were considered non-clinically significant. We cannot discard that, as suggested for CDC administration [48], a minor fraction of pCPC could be partially entrapped in the capillaries (undetectable by angiography) resulting in a focal acute inflammatory injury. However, our safety studies in healthy, non-infarcted swine do not support this possibility. A transient, mild, local immune reaction in the heart, without histologically evident rejection or systemic immunogenicity, has been described with allogeneic CDC in the rat model [6]. Alternatively, these altered plasma values could be also partly attributed to pCPC’s intrinsic secretion capacity of chemokines, growth factors, and cytokines. The role of these factors could be to promote angiogenesis, cell survival, and the proliferation and differentiation of cardiac precursors. CPC comparative secretome profile has been recently defined, and TNF-α and IL-1β are highly preferentially secreted by CPC. In the case of IL-6, it has been found to be secreted at high levels (~ 20 ng/mL) [14]. Moreover, recent studies have shown that IL-6 is produced by all major sub-populations within cardiac explant-derived stem cells, including those cell subsets with a c-Kit+ phenotype [49]. Therefore, administration of high doses of pCPC could contribute to a transient increment of circulating cytokines.

A recent meta-analysis of preclinical studies using only cardiac-derived progenitor cells has reported an overall effect of 10.7% improvement in LVEF compared with placebo [50]. As expected [51], when considering only large animal studies, CPC therapy elicited only a mean 5.2% (95%CI 3.4–7.1) improvement in LVEF compared to control animals [50]. In our study, this difference was within range in the 25 M group and higher in the animals receiving the high cell dose (50 M group). Moreover, beneficial effects were also evidenced in ventricular volumes, which decreased over time in both groups, when comparing end study determinations with those obtained before therapy. Among CMR-derived surrogate endpoints for clinical trials, MSI (an indicator of myocardium salvage) is receiving increasing interest, as the field turns to cardioprotection. This parameter gives a reasonable estimation of the benefit of a therapy, allowing comparisons between different infarct sizes, by decreasing the interpatient variability associated with absolute infarct size measurements [47, 52]. Interestingly, MSI in the present study was increased twofold in both treated groups, compared to CON, a finding that was associated to improved functional results in the treated animals, especially in the 50 M group. Reduced fibrosis and enhanced angiogenesis were also demonstrated, but these were not accompanied by any statistically significant inter-group reduction in scar size. This could be related with the delayed administration of pCPC that, although beneficial for cell survival, could allow the early development of fibrotic signals that were not timely counteracted. Eventually, combined strategies could help to extract the maximum benefit of CPC-based treatments.

The functional and physiologic similarities between pig and human cardiovascular systems and, in particular, the similar size between pig and human hearts allows for easy extrapolation of the therapeutic dose. The studies presented showed functional improvement in infarcted pigs 10 weeks after IC delivery of 25 or 50 million of allogeneic pCPC cells, evaluated by cardiac magnetic resonance imaging and histological assessment. These results are in line with clinical trial data using a similar dose of 25 million, if autologous, cardiac-derived progenitors [53]. The results reported in the present work, as well as other clinical considerations, set the ground for CAREMI clinical trial design and approval [23], as a double-blind, controlled, randomized (2:1), and multicenter I/II trial, with a dose-escalation phase. The target dose for CAREMI was fixed at 35 × 10^6^ allogeneic human CPC. This dose is within the efficacy range tested in the present study in a homologous model (pCSC in swine), conservative in terms of safety, and proven to be safe in non-infarcted animals.

In this field, the concept of allogeneic-driven benefit has been recently introduced, and initial results are encouraging [6, 54], suggesting that these cells might have advantages compared with their autologous counterparts. Consolidation of the concept would be critical for off-the-shelf product development in cellular cardiomyoplasty. ALLSTAR (NCT01458405) [22] and CAREMI (NCT02439398) [23] are the first clinical trials addressing the evaluation of allogeneic CDC and CPC, respectively, in the context of adults who have experienced a large heart attack with residual cardiac dysfunction (LVEF ≤ 45%). These trials have both reported very good safety profiles but have failed to establish efficacy. Data on 1-year follow-up from CAREMI demonstrated that CPC were well tolerated during the acute and sub-acute phases of infarct, with no immune-related adverse event reported. In addition, low (and clinically irrelevant) levels of donor-specific antibodies anti-HLA were only found in a minority of patients (6.4%). Concerning inflammation status, a significantly greater reduction in C-reactive protein levels, up to 1 month after CPC transplantation, was reported. However no statistically significant difference in infarct size, as the only end-point, was found. In order to demonstrate any positive efficacy result, the authors propose that further studies should first evaluate the optimal administration guidelines (including higher doses of CPC or multiple administrations) and focus on patients with better-identified risk of adverse remodeling [24]. On a similar note, available ALLSTAR interim results regarding phase II primary efficacy endpoints (% of change in infarct size as measured with cMRI from baseline to 12 months) showed no significant change in scar size between 6 and 12 months. Considering that the probability that any effect would be observed at 12 months was very low, all patients were transitioned to annual follow-up. In this case, the investigators suggested to look at the effects of matched versus unmatched cells [55].

Another approach that has been proposed is the combination of MSC and CSC, which in the case of the CONCERT-HF trial were autologous cells and administered transendocardially (NCT02501811) [56]. One cannot ignore, however, the turmoil in the field of cardiovascular cell therapy following the retraction of over 30 studies that were the basis for some of the early clinical trials [57]. The repercussions are severe and include, among others, an expression of concern having been issued regarding some results published in high-level scientific journals [58], the NHLBI deciding to pause the above-mentioned CONCERT-HF trial, specifically citing concerns on the scientific validity of the ckit+ literature [59], along with a widespread loss of public confidence and a call for better science. In our opinion, this should be construed as an opportunity to improve, rather than a setback, to conduct better science and, as has been suggested recently, increase studies in large animal models that, like our own, more closely mimic the clinical in vivo scenario [57] before jumping to the clinical arena. Proof of concept large animal studies are, in this setting, vital. These studies may be technically demanding, expensive, or complex, but at the same time, they are essential to not only improve clinical outcomes, but also to justify the risks and costs inherent to clinical trials [60].

The limitations of this study are related to the use of healthy, young swine to model myocardial infarction, while the typical patient with this condition is older and presents with co-morbidities and risk factors that affect the response to any therapy. We did not conduct any arrhythmia testing in this study, so we cannot discard that the animal from the 25 M group that died 5 weeks after AMI had a fatal arrhythmia. However, several previous studies have reported no arrhythmogenicity by cardiac-derived cell products [5, 15], so we do not consider it likely. Moreover, the CAREMI trial did not find any substantial arrhythmogenic event, among the 55 recruited patients, 6 months after treatment [23, 24]. In addition, the follow-up of the experiments was short of necessity. Despite being kept on a restricted calorie diet, farm swine growth rate prevents longer CMR-based studies, since the animals do not fit in the magnet bore. Another limitation is that we did not examine the production of extracellular vesicles by pCPC. However, exosomes from CDCs have been previously evaluated in an acute and chronic porcine myocardial infarction [61]. Based on that, it is expected that pCPC cells will also release exosomes, but we did not specifically look into them.

## Conclusions

Taken together, the results indicate a dose-dependent benefit of the administered cells (allogeneic pCPC; 1 week after experimental myocardial infarction) on global cardiac function. pCPC-treatment of infarcted animals prevents cardiac remodeling preserving heart function, as indicated also by the greater MSI. This effect is associated with a reduced extension and severity of fibrosis, facilitating also mature angiogenesis. No effect on inflammatory infiltration, degree of necrosis, or calcification was demonstrated. Preclinical safety and efficacy results supported the CAREMI clinical trial.

## Additional file

Additional file 1: Detailed methodology and supplementary data. **Figure S1.** Extended characterization of pCPC. **Figure S2.** Engraftment and anatomopathological analysis of pCPC transplanted hearts. **Table S1.** Plasma cytokine levels before and 24 h after each treatment. (ZIP 771 kb)

## Abbreviations

^18^F-FDG: 2-Fluoro-2-deoxy-d-glucose [^18^F]
AAR: Area at risk
CDC: Cardiosphere-derived cells
CK-MB: Creatine kinase MB
CM: Conditioned medium
CMR: Cardiac magnetic resonance
CPC: Human cardiac progenitor cells
CSC: Cardiac stem cells
cTnI: Cardiac troponin I
EDVi: End-diastolic volume indexed to body surface area
ESVi: End-systolic volume indexed to body surface area
FIS: Final infarct size
HLA: Human leukocyte antigen
IC: Intracoronary
IV: Intravenous
LAD: Left anterior descending coronary artery
LAO: Left anterior oblique
LV: Left ventricle
LVEF: Left ventricular ejection fraction
MI: Myocardial infarction
MSC: Mesenchymal stem cells
MSI: Myocardial salvage index
pCPC: Porcine cardiac progenitor cells
PET/CT: Positron emission tomography/computed tomography
pMSC: Porcine mesenchymal stem cells
qPCR: Quantitative polymerase chain reaction
RNAseq: Whole transcriptome shotgun sequencing
RT-qPCR: Quantitative reverse transcription coupled to PCR
STEMI: ST-segment elevation myocardial infarction
TIMI: Thrombolysis in myocardial infarction
