# Supplementary material for: Dose-dependent improvement of cardiac function in a swine model of acute myocardial infarction after intracoronary administration of allogeneic heart-derived cells
Source: Stem Cell Res Ther. 2019 May 31;10:152. doi: 10.1186/s13287-019-1237-6 (PMC6544975; doi:10.1186/s13287-019-1237-6)
Supplement: Supplementary file 1 — Detailed methodology and supplementary data. Figure S1. Extended characterization of pCPC. Figure S2. Engraftment and anatomopathological analysis of pCPC transplanted hearts. Table S1. Plasma cytokine levels before and 24 h after each treatment. (ZIP 771 kb) [file 13287_2019_1237_MOESM1_ESM.zip › Supplementary material scrt.docx]

**Additional file 1**

**Dose-dependent improvement of cardiac function in a swine model of acute myocardial infarction after intracoronary administration of allogeneic heart derived cells**

*Veronica Crisostomo PhD^1,2*^*†*, Claudia Baez PhD^1,2^*†*, José Luis Abad PhD^3^, Belén Sanchez BC^3^, Virginia Alvarez^3^, Rosalba Rosado^3^, Guadalupe Gómez-Mauricio PhD^1^, Olivier Gheysens^4^, Virginia Blanco PhD^1,2^, Rebeca Blazquez PhD^1,2^, José Luis Torán^5^, Javier G. Casado PhD^1,2^, Susana Aguilar^5^, Stefan Janssens MD, PhD^4^, Francisco M. Sánchez-Margallo PhD^1,2^, Luis Rodriguez-Borlado PhD^3^, Antonio Bernad PhD^5^, Itziar Palacios PhD^3*^*

1.- Fundación Centro de Cirugía de Mínima Invasión Jesús Usón. Cáceres, Spain

2.- CIBERCV. Instituto de Salud Carlos III. Madrid, Spain.

3.- Coretherapix S.L.U./Tigenix Group Madrid, Spain

4.- Department of Cardiovascular Medicine, University Hospitals and KU Leuven. Leuven, Belgium.KUL

5.- Department of Immunology and Oncology, Spanish National Center for Biotechnology (CNB-CSIC), Madrid, Spain.

† Equal Contribution

**Supplementary Methods**

1. **Isolation and culture of pCPC**

Porcine CPC (pCPC) isolation and culture were performed by Coretherapix SLU as previously reported (1) and covered by patent (2). Briefly, starting material (~1 g of cardiac tissue) obtained from healthy male Large White swine hearts was digested by combining mechanical dissociation with enzymatic digestion (collagenase type 2 (Worthington Biochemical Corporation)) to obtain a cell suspension. Cell suspension was immunodepleted of CD45-positive cells and immunoselected for CD117 (c-kit) using magnetic microbeads coupled with specific antibodies (Miltenyi Biotech). The resulting cells were seeded on pig gelatin (Sigma-Aldrich) coated plates and cultured in low O2 conditions (3%) using isolation medium (DMEM/F12 with 10% FBS, L-Glutamine, Penicilline-Streptomycine, Insulin-Transferrin-Selenium (ITS) and bFGF, IGF-II, EGF and hEPO recombinant growth factors (Invitrogen™, Peprotech and Sigma-Aldrich)). One week after seeding, the medium was replaced by growing medium (DMEM/F12 and Neurobasal medium (1:1) supplemented with 10% FBS, L-Glutamine, Pen-Strep, ITS, growth factors (bFGF, IGF-II, EGF) together with B27, N2 and β-mercaptoethanol (Invitrogen™). Cells were expanded over 3 passages and then cryopreserved in medium with 5% of DMSO. Cells were then thawed and seeded for a second expansion to obtain the finished product used in the non-clinical experiments.

1. **Extended characterization of pCPC; phenotypic and functional analysis**

Extended characterization was done in pig CPC, at passage 4 and 8 by flow cytometry and qPCR techniques. Human CPC, and MSC isolated from Bone Marrow (BM-MSC) and Adipose tissue (ADSC) were used for comparison

- 1. Analysis of cell surface markers by flow cytometry

FACS analysis was done in different batches of pCPC (attached cells) after isolation. Cells were detached from culture dishes with 0.25% trypsin-EDTA, and both were blocked with staining buffer and 1% human serum before antibody addition. Cells were incubated with antibodies or isotype matched controls for 30-40 min at 2-8ºC. The antibodies used for the characterization were showed in Supplementary 1C. Data acquisition was done using a cytometer (FACSCalibur, BD). Marker expression was analysed in viable cell (7AAD negative) gate, using CellQuest Pro (BD) software. Percentage of surface protein positive cells was calculated by marker placed to give 1% positive events for isotype control.

- 1. Gene expression analysis by real-time quantitative PCR (RT-qPCR)

Total RNA extraction of samples was performed using the Qiagen RNeasy Mini kit. The RNA extracted was analysed in a Nanodrop spectrophotometer to confirm its quantity, purity and quality. Subsequently, Promega DNase I was used for the digestion of the contaminating genomic DNA. After this procedure, the cDNA synthesis was carried out using 1 µg of RNA through Invitrogen’s SuperScript III reverse transcriptase. For each gene expression assay, 200 ng of cDNA were used as template to perform the real-time quantitative PCR with TaqMan probes for the specific genes: PECAM1: Ss03392600_u1, GATA4: Ss03383805_u1, GATA6: Ss03384121_u1). Gene expression was normalized to a housekeeping gene expression, B2M (Ss03391154_m1) and the expression of those genes was quantified relatively with respect to the BM-MSC sample using the comparative Ct analysis method (ΔΔCt).

RT-qPCR analysis for F11R and CACNG7 expression was performed using Power SYBR Green reagents (Applied Biosystems). Cycle conditions were 95°C for 10 min, followed by 40 cycles of 95°C for 15 s and 60°C for 1 min. Quantified values were normalized to GAPDH.

| Primer | Sequence (5´-3´) |
| --- | --- |
| F11R Forward | TCGAGAGGAAACTGTTGTGC |
| F11R Reverse | GAAGAAAAGCCCGAGTAGGC |
| F11R Forward (swine) | TCTTGTGCTCCCTGACGTTG |
| F11R Reverse (swine) | AATTTCCACTCCACACGGGG |
| CACNG7 Forward (h & s) | TAAAGAACCAAGCCCACCAC |
| CACNG7 Reverse (h & s) | TCAGCCTCTTCCTCGTGTTC |

- 1. Secretome Analysis by ELISA technique.

Comparative (CPC vs. pCPC) limited secretome evaluation was carried out essentially as previously described (2). pCPC and human CPC were seeded at 5000 cells/cm^2^ in growing medium with 10% serum. Next day, culture medium was replaced by FBS-free growing medium supplemented with Insulin-like Growth Factor 2 (IGF-2), basic Fibroblast Growth Factor (bFGF) and Epidermal Growth Factor (EGF). Supernatants were collected after 3 days, debris were removed by centrifugation at 3000xg for 3 min, and the concentrations of Monocyte Chemoattractant Protein 1 (MCP-1 or CCL2), Hepatocyte Growth Factor (HGF), Transforming Growth Factor β1 (TGF-β1), Vascular Endothelial Growth Factor (VEGF) and Insulin-like Growth Factor 1 (IGF-1) and were measured with ELISA kits (R&D Systems Inc., Minneapolis, MN), according to the manufacturer’s instructions. Given the limited number of porcine-specific ELISA kits, both swine and human supernatants were measured using human-specific ELISA kits.

- 1. Transwell migration assay

Migration of MonoMac-1 cell line (DSMZ) was determined by flow cytometry in Boyden chambers with 5µm pores. Porcine and human CPC were seed at 10000cells/cm^2^ in culture medium for 24h then medium was replacement by growing medium without FBS and Factors. Cell culture supernantants were collected after 5 days and used as stimuli in the lower compartment of the transwell chambers. MonoMac-1 were plated in the upper compartment and incubated for 4h at 37ºC with 5% CO2. The number of migrated MonoMac-1 (into the lower compartment) was counted by flow cytometry using a fixed sample volume and a fixed acquisition time. To normalize and standardized the counts a fixed amount of fluorescent microbeads of know concentration (Flow cytometry Absolute Count Standard Beads, Immunostep, Spain) were used. The migration index was calculated as follow: ratio between the number of migrated cell in response to different stimuli (mean of the duplicates less background one) and the cells migrated in the absence of stimulus (background).

- 1. Karyotype analysis

The karyotype of pCPC at P8 was analyzed. Cells were subjected to hypotonic treatment and fixed, and the chromosome numbers were counted from 100 spreads under an oil immersion objective upon Giemsa staining.

1. **Detection of Y chromosome sequences**

For the detection of male-derived pCPC or their progeny in female swine hearts, Y-chromosome PCR was carried out from tissue samples obtained from the transition area in all hearts. DNA extraction was performed with TRI Reagent (Sigma, St. Louis, MO, USA) according to the manufacturer's instructions. The detection of male cells in a female recipient was carried out by PCR using the Taq DNA Polymerase (Invitrogen, Carlsbad, Ca). Amplification consisted in 40 cycles of 30 s at 94°C for melting, 30 s at 55°C for annealing and one min at 72°C for amplification, using the primers (Table S) designed to allow the amplification of Y-chromosome specific sequences (NCBI Reference Sequence: NC_010462.2).

1. **Large animal model experimentation**

The study protocols were approved by the Institutional Animal Care and Use Committee, and complied fully with the Guide for the Care and Use of Laboratory Animals: Eighth Edition (National Research Council. Washington, DC: The National Academies Press, 2010).

A total of 40 (28 infarcted for dose-response; 6 healthy swine for acute safety and 7 infarcted swine for biodistribution analysis) female Large White swine, weighing 30 - 34 kg, were included in the study for large animal models. Since pCPC were of male origin, only female swine were included in the study in order to use Y-chromosome determination to look for the transplanted pCPC or their progeny.

1. Anaesthesia and monitoring protocols

After being fasted for 24 hours, animals were premedicated by the intramuscular injection of ketamine (20 mg/kg). 10 minutes after premedication, access to an ear’s marginal vein was obtained and anaesthesia was induced with intravenous (IV) 1% propofol (3 mg/kg). Endotracheal intubation was performed using cuffed endotracheal tubes (sizes 6.5 - 9, depending on the animal’s weight).

For MR examinations, anaesthetic maintenance was performed with a continuous propofol infusion (8-12 mg/kg/h). Animals were connected to a MR-compatible ventilator (TransPAC T200, Smiths Industries Medical Systems, UK) and mechanical pressure controlled ventilation was established with a FiO2 of 0.5 to assure normocapnia.

During infarct induction and pCPC administration, anaesthesia was maintained using a continuous IV infusion of a combination of 1% propofol (10-12 mg/kg/h) and remifentanyl (15-18 μg/kg/h). Endotracheal tubes were connected to a semi closed circular anaesthetic circuit attached to a ventilator (Leon Plus, Heinen & Löwenstein, Bad Ems, Germany) with a fresh gas flow rate of 1 L/min (0.4/0.6 mixture of oxygen and air). Controlled ventilation was established with a tidal volume of 6-8 mL/kg to obtain normocapnia (with a CO2 pressure of 35-40 mmHg). Lidocaine (Lidocaína 2% Braun, B/Braun) was administered continuously at a rate of 1 mg/kg/h.

Systemic heparin was injected IV (150 IU/kg) 5 minutes prior to percutaneous sheath placement.

Anaesthetic monitoring included cardiovascular and hemodynamic parameters such as: heart rate, electrocardiography, pulse-oximetry and invasive arterial blood pressure. Ventilatory parameters registered were: respiratory rate, oximetry, airways pressure, inspired and end-tidal CO_2_ concentration.

Once the follow-up was completed, animals were euthanized by a lethal dose of potassium chloride (1-2 mmol/kg) while under deep anaesthesia, as recommended by the American Veterinary Medical Association (AVMA Guidelines for the Euthanasia of Animals: 2013 Edition. Available at: https://www.avma.org/kb/policies/documents/euthanasia.pdf).

1. Acute Myocardial Infarction in Large White swine

Animals were subjected to a complete physical examination and serum biochemical assay before being included in the protocol. Premedication was performed with 400mg oral amiodarone daily from 5 days before to 3 days after model creation and with 500mg aspirin and 300mg clopidogrel on the day before infarction. Aspirin (500mg and clopidogrel (75mg) administration were maintained through the duration of the study. In all cases, animals underwent a baseline CMR prior to infarct creation. Then a MI was induced through a percutaneous femoral approach by a 90-min occlusion of the LAD immediately distal to the origin of the first diagonal branch, as previously described^1^. For this purpose, a 7Fr introducer sheath (Terumo, Inc. Tokyo, Japan) was placed percutaneously in the right femoral artery using the Seldinger technique and a 6 Fr hockey stick guiding catheter (Mach 1 ®, Boston Scientific Corporation, Natick, MA, USA) was navigated to the origin of the left coronary artery under fluoroscopic guidance and 150 µg of nitroglycerin were injected through it to prevent coronary spasm. A coronary angiogram was acquired in the 40º left anterior oblique (LAO) projection and a coronary bare metal stent (Apolo, Iberhospitex, SA, Spain) of appropriate size was placed over a 0.014” guidewire (Hi-torque. Abbott Vascular, Santa Clara, CA, USA) immediately below the origin of the first diagonal branch. The deploying balloon was kept inflated for 90 minutes. Total LAD occlusion was assessed by contrast injection through the guiding catheter immediately after balloon inflation and just before deflation. After this time, the balloon was deflated, coronary patency checked and catheters and sheath removed. Hemostasia of the puncture site was achieved by manual compression. Anesthesia and lidocaine infusion were maintained for another hour with the aim of treating possible arrhythmias during the reperfusion period. In case of ventricular fibrillation either during occlusion or after reperfusion, manual chest compressions and 200 J biphasic defibrillation shocks (Zoll M series biphasic 200J, Zoll Medical Corporation, Massachusetts, USA), and pharmacological therapy when needed were used to revert them.

Postoperative analgesia was obtained with 10 µg/kg/12h of IM buprenorphine during the first 24h. A fentanyl transdermic release patch (25 μg/h) was used to assure correct analgesia in the immediate postoperative period. Prophylactic antibiotics were administered in all cases for 5 days after each procedure (ceftiofur hydrochloride).

1. Group allocation and Intracoronary pCPC administration.

One week after MI induction, surviving animals were included in the study. A second CMR study was conducted and animals were sequentially allocated to receive 25x10^6^ pCPC (25M group, n=11), 50x10^6^ pCPC (50M, n=7) or vehicle (CON, n=7). In lieu of randomization, CMR processing was not performed before group allocation to avoid selection bias.

The administration solution, or vehicle, was obtained by mixing physiologic saline (Braun Medical SA, Spain) with human serum albumin (Grifols, Spain) to a final concentration of 5%. The day of the treatment, cells were thawed at 37ºC and suspended in 20mL of vehicle. A cell count was performed and the concentration of the suspension was adjusted to 2.1x106 cells/mL and 12, 18 or 24 mL of cell suspension for 25x10^6^, 35x10^6^ and 50x10^6^ cell doses, respectively, were delivered intracoronary. Animals treated with vehicle received 24 mL of administration solution. Full allocation concealment could not be performed due to the different volumes being administered, so, as explained below, CMR processing was deferred until all experimental procedures had been completed.

Once in the angio-suite room, access to the LAD was established as described for infarct creation, a coronary angiogram was obtained to assess flow in the LAD (TIMI score) and a 3 Fr microcatheter (Microferret infusion catheter, Cook Medical. Bloomington, IN, USA) was navigated to the level of the coronary stent. The microcatheter was attached to a micro-aggregate filter, where the syringes with the treatment product were connected. The total volume to be infused was divided in 6mL syringes, and administered manually at 2mL/min followed by a 3 min rest period to allow for cell extravasation. Injection cycles were repeated to the total dose depending on the group. Administration was performed without stop-flow conditions. A completion coronary angiogram was acquired 5 min after administration to assess coronary TIMI grade flow again. The femoral sheath was then removed and hemostasia of the puncture site achieved by manual compression.

1. Assessment by Cardiac Magnetic Resonance

Cardiac Magnetic Resonance (CMR) was performed for morphological and functional assessment before injection (day 0), and at one and 10 weeks after pCPC/vehicle infusion. In all cases, animals were placed inside the MR system (Intera 1.5 T, Philips Medical Systems. Best, The Netherlands) in the sternal decubitus. Retrospective cardiac triggering was used. A 4 elements phase array coil was placed around the animals’ chest. Images were acquired in the intrinsic cardiac planes: short axis, vertical long axis and horizontal long axis views. For measurement of left ventricular function and mass breath hold gradient echo cine images were obtained over the entire left ventricle (LV). Typical parameters used were: slice thickness: 8 mm, no gap, Field of view (FOV): 320 x 320 x 80, matrix: 192x192, flip angle: 60°, repetition time/echo time (TR/TE): 4.4/2.2. For edema visualization and area at risk measurements, three short axis slices were acquired (basal, mid-heart and apical slices) using a T2 weighted short tau inversion recovery black blood sequence (STIR. Slice thickness 7mm, FOV:350x350, TR 2.0s, TE 80ms). For infarct size measurements, short axis images were acquired 5 to 15 min after the injection of 0.2 mmol/kg of a gadolinium based contrast agent (Gadobutrol. Gadovist 1.1 mmol/l, Bayer Schering Pharma AG, Berlin, Germany) using a breath-hold 3D gradient-echo inversion-recovery sequence. Inversion time was chosen for each sequence using a Look-Locker sequence and selecting the time that yielded the best nulling of the myocardial signal, which typically ranged from 150 to 190 ms. Imaging parameters used for the delayed enhancement images were slice thickness: 8 mm, no gap, FOV: 330 x 330 x 50, matrix: 224x200, flip angle: 15°, TR/TE: 4.9/1.67.

Once all animals had completed follow-up, CMR images were analyzed independently by two researchers blinded to group allocation using commercially available software (Intellispace Portal 7.0.1.20482 Philips Medical Systems. Best. The Netherlands). For these analyses, endocardial and epicardial borders were manually delineated in end diastolic and end systolic short axis views, in all slices, and the following LV functional parameters were calculated: end diastolic volume (EDV), end systolic volume (ESV) and ejection fraction (EF). Ventricular volumes were indexed to body surface area to offset the effect of the animals’ growth on the results. Area at risk was calculated on the examination performed before pCPC injection by defining normal and edematous myocardium with computer assistance on the mid-heart slice. Similarly, infarct size calculations were performed in the delayed enhancement images by manually defining the normal and infarcted myocardium with computer assistance to obtain the percentage of infarcted left ventricle. Central dark zones within the area of edema/hyperenhancement were included. Myocardial Salvage Index (MSI) was then calculated as area-at-risk (AAR) at mid-heart slice minus final infarct size (FIS) in an equivalent slice divided by area at risk (MSI=(AAR-FIS)/AAR).

1. PET/CT acquisition and analysis

Animals (n=7) were imaged on a Biograph mCT PET scanner (Siemens Medical Solutions, Hoffman Estates, IL, USA) 4 hours after cell administration. A detailed description of this PET/CT system and its performance characterization can be found elsewhere (4). This scanner allows point spread function depth-dependent resolution recovery and time-of-flight acquisition and reconstruction (5). Visual analysis of the pCPC biodistribution was performed using a workstation with True D software (Siemens Healthcare, Erlangen, Germany). For the quantitative analysis, all studies were exported and analyzed using the PMOD software (PMOD Technologies Ltd., Adliswil, Switzerland).

The acquisition protocol consisted of a whole-body CT scan for attenuation correction and a 3D whole-body PET emission scan (5-6 bed positions, 10 minutes per bed position). Images were reconstructed with the 3D-OP-OSEM+PSF algorithms with TOF with 3 iterations and 21 subsets respectively (matrix sizes 200 × 200 and a Gaussian post- reconstruction ﬁlter with FWHM = 2 mm).

A total-body volume of interest (VOI) was drawn to measure the total activity in the body, expressed in KBq. Additional VOIs were drawn over heart, lungs, liver, spleen, kidneys and bladder. All the VOIs were created over CT images and then copied and pasted onto the corresponding PET images. The percentage of injected dose in the organs (%ID) was then calculated as the ratio of activity in the organ VOI and activity in the total-body VOI. Afterwards, considering the physiological distribution of ^18^F-FDG (6) the percentage of cell retention in the heart was calculated assuming that the signal produced by the cells was located exclusively at the heart and the lungs.

1. Blood chemistry determinations

Blood samples were taken at baseline and 24 h after infarction, before and after cell or vehicle infusion and at sacrifice to measure cardiac troponin as cardiac injury marker and cytokines as indicators of inflammation. cTnI was measured by immunoassay using a commercially available system (AQT90 Flex, Radiometer Iberica SL, Madrid, Spain).

For further safety determination, plasma cytokines were studied in a subset of pigs (n=12, 4 belonging to each group) in samples obtained before and 24 hours after pCPC administration. For this purpose, all plasma samples were stored at -20 ºC until use and processed together. After thawing, IFNα, IFNγ, IL-1β, IL-4, IL-6, IL-8, IL-10, IL-12p40 and TNFα were quantified using a multiplexed immunoassay. All measurements were performed according to the manufacturer’s instructions by Luminex xMAP technology using the ProcartaPlex Porcine Cytokine & Chemokine Panel (eBioscience, San Diego, CA, USA). The concentrations of the different cytokines were calculated according to a standard curve and expressed as pg/mL.

1. Histopathological analysis

Samples were then taken from the infarct core and infarct border areas, fixed in 4% formalin and processed for hematoxylin-eosin and Masson’s trichromic stains using standard techniques. A scoring system ranging from 0 (absent or normal) to 4 (severe) was used to grade the presence of inflammatory infiltrate, fibrosis, necrosis, calcification or teratoma formation. For the evaluation of angiogenesis at the infarct border, the density and size of blood vessels were determined from five randomly chosen areas of each sample, as previously described (7).

**References**

1. Crisostomo V, Baez-Diaz C, Maestre J, Garcia-Lindo M, Sun F, Casado JG, et al. Delayed administration of allogeneic cardiac stem cell therapy for acute myocardial infarction could ameliorate adverse remodeling: experimental study in swine. J Transl Med. 2015;13(1):156.

2. Luis Rodriguez-Borlado, Itziar Palacios, José Luis Abad, Belén Sánchez, Virginia Álvarez and Rosalba Rosado. Adult cardiac stem cell population. [Coretherapix Slu](http://www.google.com/search?tbo=p&tbm=pts&hl=en&q=inassignee:%22Coretherapix+Slu%22) 2016; US 20140271575 A1.

3. Toran JL, Aguilar S, Lopez JA, Torroja C, Quintana JA, Santiago C, et al. CXCL6 is an important paracrine factor in the pro-angiogenic human cardiac progenitor-like cell secretome. Sci Rep. 2017;7(1):12490.

4. Jakoby BW, Bercier Y, Conti M, Casey ME, Bendriem B, Townsend DW. Physical and clinical performance of the mCT time-of-flight PET/CT scanner. Phys Med Biol. 2011;56(8):2375-89.

5 Marti-Climent JM, Prieto E, Dominguez-Prado I, Garcia-Velloso MJ, Rodriguez-Fraile M, Arbizu J, et al. [Contribution of time of flight and point spread function modeling to the performance characteristics of the PET/CT Biograph mCT scanner]. Rev Esp Med Nucl Imagen Mol. 2013;32(1):13-21.

6. Fanti S, Farsad M and Mansi *L,* Atlas of PET-CT. A quick guide to image interpretation*, Verlag Berlin Heidelberg, Springer, 2009, pp. 288*

7. Schuleri KH, Amado LC, Boyle AJ, Centola M, Saliaris AP, Gutman MR, et al. Early improvement in cardiac tissue perfusion due to mesenchymal stem cells. Am J Physiol Heart Circ Physiol. 2008;294(5):H2002-11.

**Supplementary Figures Legends**

**Figure S1: Extended characterization of pCPC** **A.** Representative karyotype of pig cardiac progenitor cells (1000×) after 8 passages in culture. The pig chromosome number was 2n = 38. The sex chromosome type was XY. No changes were observed in the karyoptype. **B.** Percentage of positive cells in different pCPC isolates (01, 05, 06, 08), compared with pMSC (from BM, BM-MSC or adipose tissue, ADSC).

**Figure S2. Engraftment and anatomopathological analysis of pCPC transplanted hearts. A.** Figure shows spiking of several amounts of male cells in 10^6^ female cells. Diagnostic fragment (250 bp) was estimated to have a detection sensitivity of 1:1000. Recipient swine (all female) were IC transplanted with 25-50x10^6^ pCPC (or placebo control group), 1 week after infarction induction. Then, 10 weeks after treatment, at sacrifice heart samples were obtained from the infarct border and genomic DNA was evaluated by PCR for the presence of Y-chromosome specific sequences. Figure shows the analysis of representative animals of each group, showing no (or under the sensitivity threshold) Y-chromosome sequences. **B.** Histopathologic score. Inflammatory infiltrate, fibrosis, necrosis, calcification and teratoma were evaluated in 3 samples from the infarcted area per animal. Score was defined as: Absent=0, slight=1, mild=2, moderate=3, severe=4 for each of the pathologic characteristic in hematoxilin–eosin stained sections.

**Table S1: Plasma cytokine levels before and 24 h after each treatment.**

|  | **CON (n=4)** | | **25M (n=4)** | | **50M (n=4)** | |
| --- | --- | --- | --- | --- | --- | --- |
|  | **Pre-adm** | **Post-adm** | **Pre- adm** | **Post- adm** | **Pre- adm** | **Post- adm** |
| **IL-1β**  **(pg/ml)** | 0.33±0.66 | 1.51±2.23 | 5.33±9.79 | 1.18±2.36 | 7.87±5.59 | 7.31±10.81 |
| **IL-6**  **(pg/ml)** | 4.26±8.52 | 4.77±9.54 | 30.54±28.28 | 53.37±48.30 | 10.71±12.37 | 41.76±55.82* |
| **TNF-α (pg/ml)** | 20.00±7.02 | 9.22±8.73 | 6.86±5.92 | 6.86±5.92 | 3.62±7.24 | 65.14±99.99* |
| **cTnI**  **(µg/L)** | 0.29±7.31 | 0.27±0.26 | 0.05±0.05 | 0.65±1.10 | 0.12±0.15 | 0.43±0.65 |

Measurements of plasma cytokines revealed that six out of nine cytokines (i.e. IFNγ, IL-4, IL-8, IL-10 and IL-12p40) were undetectable, which could be related to the detection limit of commercially available swine immune reagents. Although a high dispersion of values was observed in each of the group of treatment a statistically significant increase was observed for IL-6 and TNF-α in the animals treated with 50 M of pCPC.
