# Supplementary figures and images for: Dose-dependent improvement of cardiac function in a swine model of acute myocardial infarction after intracoronary administration of allogeneic heart-derived cells
Source: Stem Cell Res Ther. 2019 May 31;10:152. doi: 10.1186/s13287-019-1237-6 (PMC6544975; doi:10.1186/s13287-019-1237-6)

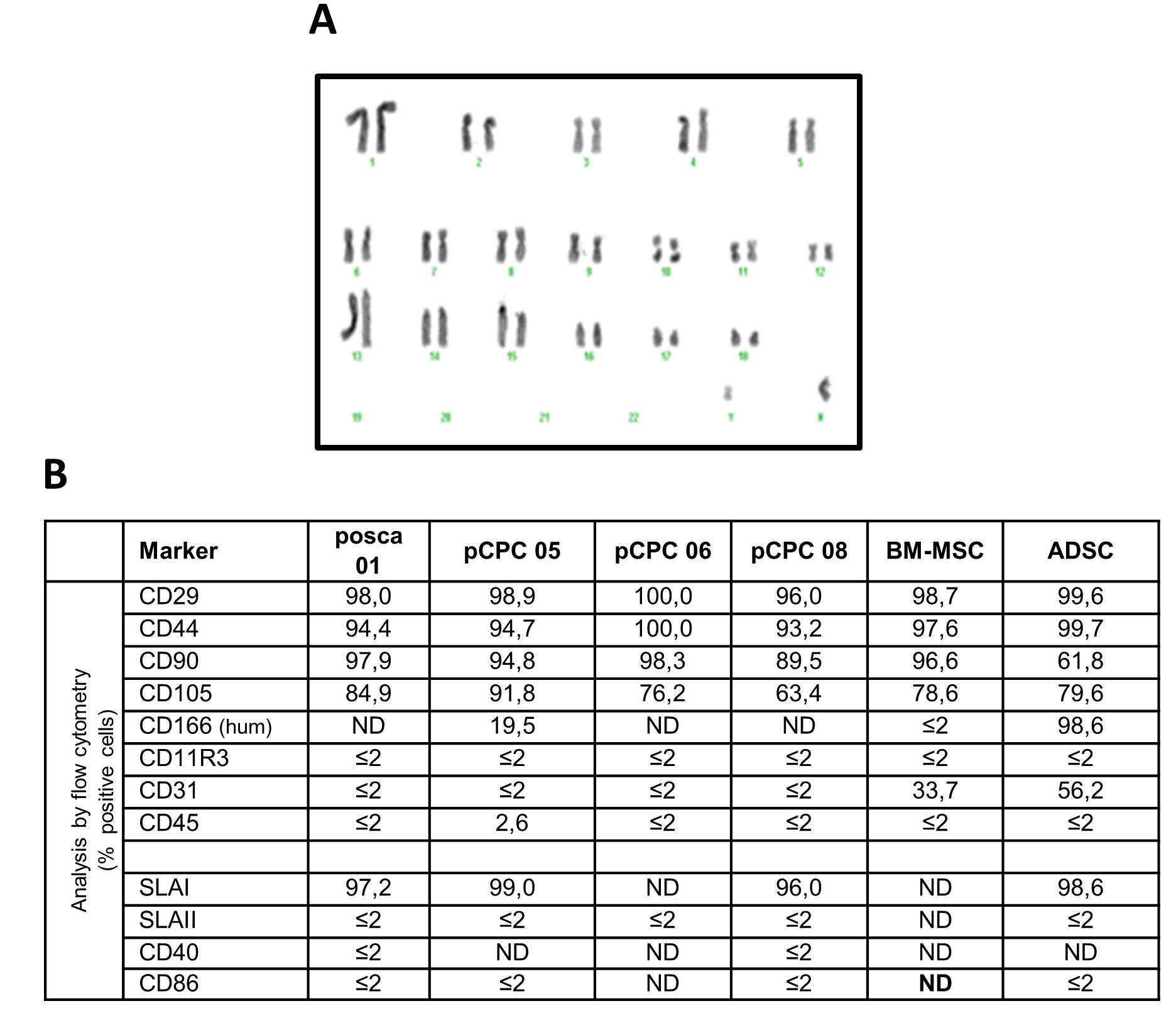

Supplement: Supplementary file 1 — Detailed methodology and supplementary data. Figure S1. Extended characterization of pCPC. Figure S2. Engraftment and anatomopathological analysis of pCPC transplanted hearts. Table S1. Plasma cytokine levels before and 24 h after each treatment. (ZIP 771 kb) [file 13287_2019_1237_MOESM1_ESM.zip › Supp fig 1.tif]

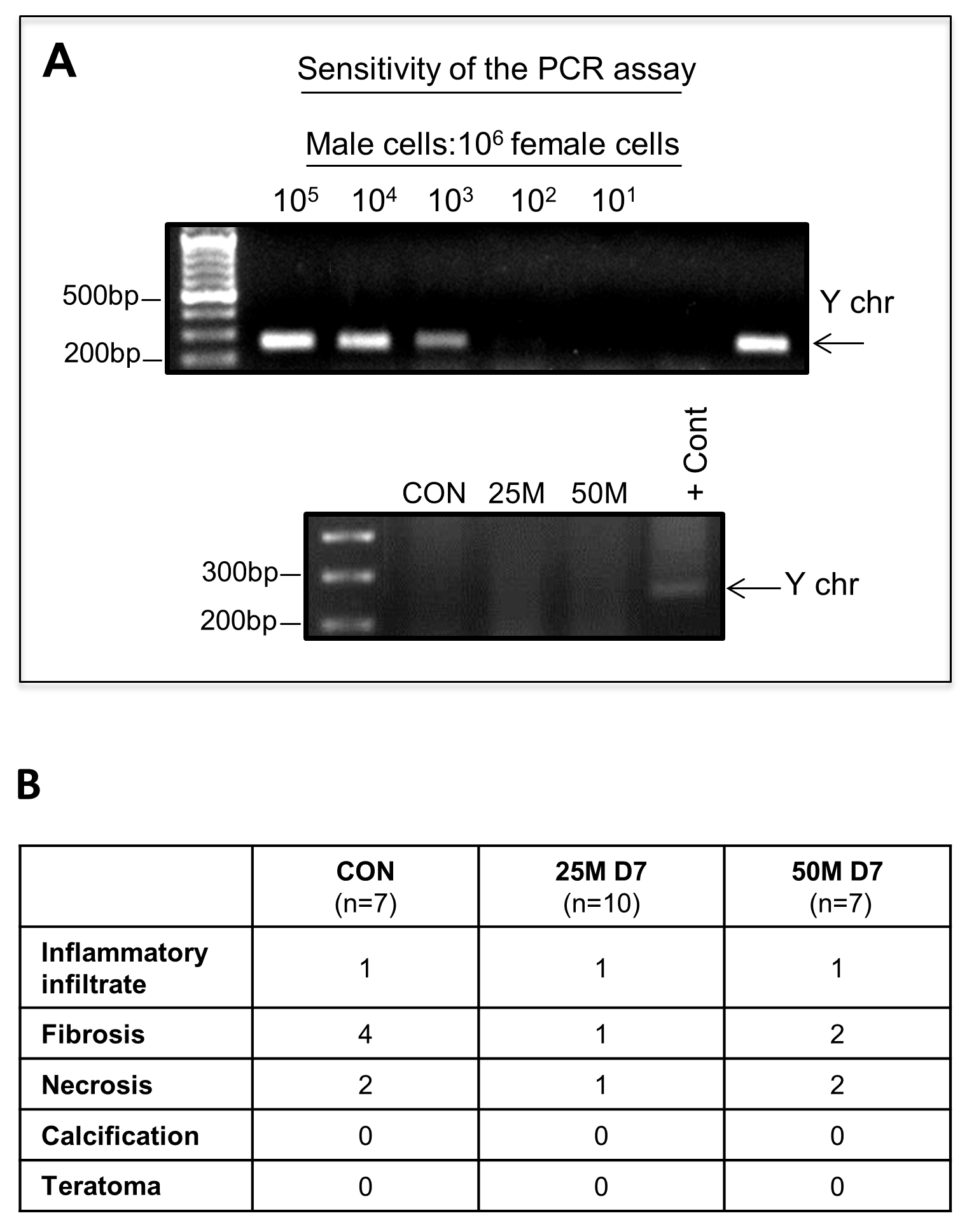

Supplement: Supplementary file 1 — Detailed methodology and supplementary data. Figure S1. Extended characterization of pCPC. Figure S2. Engraftment and anatomopathological analysis of pCPC transplanted hearts. Table S1. Plasma cytokine levels before and 24 h after each treatment. (ZIP 771 kb) [file 13287_2019_1237_MOESM1_ESM.zip › Supp fig 2.tif]
